# Modeling adaptive reversible lanes: A cellular automata approach

**DOI:** 10.1371/journal.pone.0244326

**Published:** 2021-01-04

**Authors:** Dante Pérez-Méndez, Carlos Gershenson, María Elena Lárraga, José L. Mateos

**Affiliations:** 1 Instituto de Investigaciones en Matemáticas Aplicadas y en Sistemas, Universidad Nacional Autónoma de México, Ciudad de México, México; 2 Centro de Ciencias de la Complejidad, Universidad Nacional Autónoma de México, Ciudad de México, México; 3 Lakeside Labs GmbH, Klagenfurt am, Wörthersee, Austria; 4 Instituto de Ingeniería, Universidad Nacional Autónoma de México, Ciudad de México, México; 5 Instituto de Física, Universidad Nacional Autónoma de México, Ciudad de México, México; Tongii University, CHINA

## Abstract

Dealing with traffic congestion is one of the most pressing challenges for cities. Transport authorities have implemented several strategies to reduce traffic jams with varying degrees of success. The use of reversible lanes is a common approach to improve traffic congestion during rush hours. A reversible lane can change its direction during a time interval to the more congested direction. This strategy can improve traffic congestion in specific scenarios. Most reversible lanes in urban roads are fixed in time and number; however, traffic patterns in cities are highly variable and unpredictable due to this phenomenon’s complex nature. Therefore, reversible lanes may not improve traffic flow under certain circumstances; moreover, they could worsen it because of traffic fluctuations. In this paper, we use cellular automata to model adaptive reversible lanes(aka dynamic reversible lanes). Adaptive reversible lanes can change their direction using real-time information to respond to traffic demand fluctuations. Using real traffic data, our model shows that adaptive reversible lanes can improve traffic flow up to 40% compared to conventional reversible lanes. Our results show that there are significant fluctuations in traffic flow even during rush hours, and thus cities would benefit from implementing adaptive reversible lanes.

## Introduction

One of the major challenges in modern cities is to reduce traffic congestion [1, 2]. Improving public transport and promoting the use of alternative vehicles such as bikes and scooters is critical to reducing the number of cars on the streets. In addition to the above mentioned, we can also improve traffic congestion by implementing smart routing strategies, smart traffic lights, or changes in the infrastructure, such as reversible lanes. Far from conflicting, these strategies are complementary to improve traffic congestion. In the 1920s, some cities in the United States started to implement reversible lanes to deal with increasing traffic [3]. The reversible lane approach lies in this assumption: During rush hours, traffic flow in some two-way streets increases in one direction with respect to the other direction. In most cities, it is expected that early in the morning, people living in the suburbs commute to their workplaces in the center of the city and return home in the afternoon. This behavior creates an asymmetric demand in the roads that connect the city’s border with the center. Using this insight, city planners can decide to change a lane to a more demanding direction in a specific time interval [4]. As a result, the road’s capacity is increased in the direction with more traffic flow but reduced in the opposite direction. The reversible lanes are an easy way to increase road capacity without adding new infrastructure. However, they require exceptional planning and operation to have adequate performance.

The existing methods for setting up a reversible lane are based in the observation that in most cases, there are two distinct peaks on the traffic demand, one in the morning and one in the afternoon. Based on these patterns, traffic managers decide to change the reversible lanes’ direction for a fixed time [5]. It could be one or several hours, depending on the duration of the rush hour [6]. When the traffic behaves as expected, the reversible lanes perform effectively. However, traffic in a big city is just a part of a complex system; numerous components and their interactions determine traffic flow patterns [7]. As a consequence, traffic congestion is a highly unpredictable phenomenon. It is hard to predict the traffic flow fluctuations at short time scales because it could be affected by an accident, a closed street, or faulty traffic lights kilometers away. We are now aware that reversible lanes cannot operate optimally because of these traffic flow fluctuations. We can even think of extreme scenarios where the traffic demand is the opposite of the expected one, resulting in a worse performance than the situation without any reversible lane.

Since the beginning of 2020, many urban areas worldwide have experienced massive mobility changes due to the COVID-19 pandemic. The restrictions imposed by governments and the disease itself have shaped how we move in the city [8]. Alternative transport modes, such as bikes and scooters, have more demand than before the pandemic. In contrast, public transport systems, such as the metro and bus, are in much lower demand because people are trying to avoid crowded environments. Similarly, we can expect an increase in the number of people who prefer to use a particular vehicle due to the worry of becoming infected. Once the pandemic is over, there is a high risk that those who shifted from public transport for the private vehicle never go back. This situation could have adverse consequences for mobility in cities. Recent studies have shown evidence of how the pandemic has changed our mobility habits; in particular, in [9], we see how the morning rush peak and the afternoon rush peak have been flattened. This phenomenon is a direct consequence of the increment in remote work and schools’ shutdowns, among others. The flattening of the rush peaks makes it even harder to predict the traffic patterns. As reversible lanes based their functioning in the existence of these rush hours, adaptive reversible lanes make sense for the foreseeable future.

In recent years, we are experiencing considerable changes in using data to understand and solve complex problems in almost every field. Technological advances have allowed us to generate and process real-time data through mobile phones and other connected devices to understand mobility patterns [10, 11]. Not only that, but the increasing availability of data sets and the use of social networks have revolutionized the whole field of human mobility [12–14]. These advances open the possibility to build systems that can use this information to make informed decisions in complex environments. We have also explored the coordination of traffic lights using self-organization [15–17] and the adaptive regulation of public transportation systems [18, 19]. In transportation research, some authors have studied reversible lanes as a network optimization problem [20–22] to find the optimal number and location of reversible lanes in a traffic network. In most of these studies, reversible lane allocation cannot be dynamically changed according to the traffic volume of the road, and manual operation is needed.

Some authors have proposed mechanisms to control dynamically reversible lanes. Kory Krause et al. compare different diamond design intersections with dynamic reversible lanes [23]. They identify some scenarios where the intersection with the dynamic reversible lane increases traffic flow with respect to the conventional diamond intersections. Matthew Hausknecht et al. proposed a linear programming formulation to optimize the number of reversible lanes that maximize traffic flow [24]. In [25], a bi-level programming model to optimize reversible lane assignment in signalized intersections was proposed.

Recently, some strategies to implement conventional reversible lanes safer and more efficient have been proposed. Safety impacts of roadway design have been studied in [26–29]. In [30], the authors present an automatic mechanism to separate the lanes physically without human intervention. In [31], authors propose a mechanism to control reversible lanes using real-time information.

In this work, we propose a framework based on a cellular automata model to evaluate the potential benefits of an adaptive reversible lanes versus conventional, prescheduled reversible lanes. Our framework allow us to determine analytically if specific locations can benefit from an adaptive reversible lane based on existent traffic data. We show that even during rush hours, in most cases traffic fluctuations are notorious, so an adaptive approach improves traffic flow.

Instead of having reversible lanes in a particular direction during a fixed period of time, we allow the lanes to change their direction depending on real-time traffic information. Unlike traditional reversible lanes, adaptive reversible lanes can adapt to traffic flow fluctuations improving its performance. In addition, the adaptive reversible lanes could handle different transportation modes, such as bikes or buses according to the demand and traffic conditions. In the next sections, we describe our framework and propose a methodology to model adaptive reversible lanes with cellular automata. We show that adaptive reversible lanes are more efficient than conventional reversible lanes and we quantify the traffic flow fluctuations during rush hours. Finally, we discuss potential benefits of adaptive lanes and challenges in their implementation.

## Materials and methods

### Nagel-Schreckenberg model

Nagel-Schreckenberg (NaSch) is a well known cellular automata model for freeway traffic [32]. In this model, each lane is represented by a uni-dimensional lattice of length *L*. Each vehicle has an associated *x* position on the highway. By convention, the position is increasing, starting at index 0. Each vehicle has an associated velocity *v*, which is an integer value such that: *v* ∈ {0, 1, …, *v*_*max*_}. Each cell on the lattice represents either a vehicle or space between them. The cells of the lattice are updated synchronously at discrete time steps according to the following rules:

- Acceleration. If velocity *v* of a vehicle is less than the maximum velocity *v*_*max*_ and if the distance *d* (in unit cells) to the vehicle in front is greater than *v* + 1, then, *v* = *v* + 1.
- Braking. If a vehicle in cell *i* encounters a vehicle in cell *i* + *j*(*j* ≤ *v*), then *v* = *j* − 1.
- Stochasticity. If the velocity *v* is greater than 0, *v* = *v* − 1 with probability *p*, where *p* is the probability that a vehicle will slow down randomly.
- Movement. Each vehicle moves *v* cells.

It has already been shown that variants of the NaSch model deliver precisely the same results for vehicle trajectories as kinematic wave models and linear vehicle-following models [33]. Furthermore, the NaSch model may produce realistic behavior at the macroscopic level, and the equivalency of its behavior to various macroscopic models has also been demonstrated.

### Reversible lane model

In this work, we compare conventional reversible lanes’ behavior and adaptive reversible lanes in terms of the macroscopic traffic flow based on historical data. In particular, our model aims to a self-driving car environment, where the full potential of adaptive lanes is expected to take place. For these reasons, to carry out the simulation results presented in our paper, we use the NaSch model parameters to reflect the real freeway traffic flow behavior when adaptive reversible lanes are used. Thus, we use the following parameters:

- *L* = 1000
- *v*_*max*_ = 5
- *p* = 0.5

We set each time step to be equivalent to one second. The size of each cell is 7.5 meters. With these parameters, the maximum speed is equivalent to 135*k*/*m*. We should note that the parameters we use are chosen to achieve a specific maximum speed. However, changing the time scale parameters does not affect the macroscopic behavior of the system. [34]

With the NaSch model implemented, we ran 1000 simulations to obtain mean flows for each density (*ρ*) value from 0 to 1 in 0.001 increments. We can calculate the density as *ρ* = *N*/*L*, where N is the number of cells occupied by a vehicle, and L is the size of the lattice. Density *ρ* = 0 means each cell in the lattice is empty, and *ρ* = 1, means every cell is occupied by a vehicle. Flow is defined as:

$$\begin{array}{c} q=\frac{1}{T*L}\sum_{t=1}^{T}\sum_{i=1}^{N}v_{i}\left(t\right) \end{array}$$ (1)

**Fig 1:**
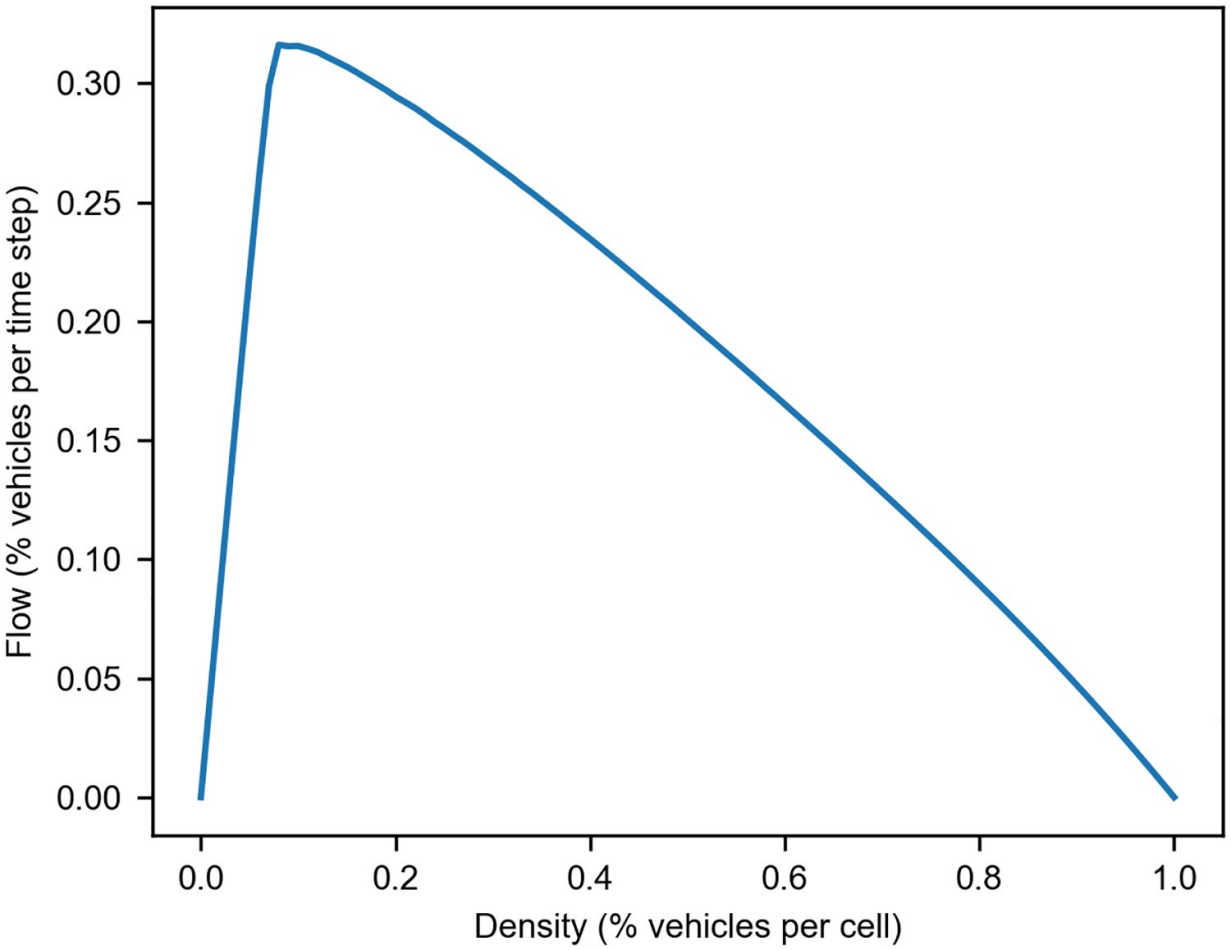
Fundamental diagram of traffic flow. We show the relation between flow and density values obtained from computational simulations. The values correspond to the average flow obtained from 1000 simulations. At lower densities, we have the freeflow phase. The higher the density on the congested phase (negative slope), the lower the flow. The maximum flow corresponds to the capacity of the lane.

**Fig 2:**
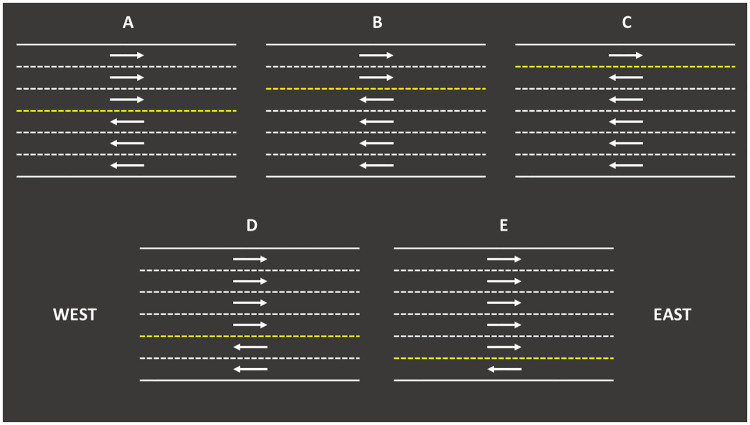
Six-lane road with adaptive reversible lanes. (A) 3-3 lane configuration. (B) 4-2 lane configuration. (C) 5-1 lane configuration. (D) 2-4 lane configuration. (E) 1-5 lane configuration.

Where, T is the number of iterations *v*_*i*_(*t*) is the velocity of the vehicle *i* at time *t*. Then we can calculate the mean flow values across all simulations for each density value and derived this fundamental diagram (see Fig 1):

With the NaSch model, we simulate a single lane road. However, our purpose is to model a road with one or more reversible lanes. To illustrate the idea, we are going to assume a six-lane road scenario. In the standard case, we have three lanes going to the east and three to the west. However, with reversible lanes, we can have five lane configurations (See Fig 2): one lane to the west and five lanes to the east (1-5), two lanes to the west and four lanes to the east (2-4), three lanes to the east and three lanes to the west (3-3, which is the standard configuration), four lanes to west and two lanes to the east (4-2) and finally, five lanes to the west and one lane to the east (5-1). We can see that configurations (1-5) and (5-1), as well as (2-4) and (4-2), are symmetrical.

From the NaSch model we derive analytically two density values. The density to the east *ρ*_*e*_ and the density to the west *ρ*_*w*_ for any of the possible lane configurations as follows:

$$\begin{array}{c} \rho_{e}=\frac{\rho *\frac{l_{w}}{l_{e}}*P_{e}}{1-P_{e}} \end{array}$$ (2)

$$\begin{array}{c} \rho_{w}=\frac{\rho *\frac{l_{e}}{l_{w}}*\left(1-P_{e}\right)}{P_{e}} \end{array}$$ (3)

Where, *ρ* is a given density between 0 and 1, *l*_*e*_, *l*_*w*_ are the number of lanes to the east and the west respectively, and *P*_*e*_ is the probability of vehicles going to the east (1 − *P*_*e*_ is the probability of vehicles going to west). This approach allows us to obtain a fundamental diagram for any lane configuration. For instance, let us say that we want to calculate the mean flow for a given density of 0.3 on a 3-3 road configuration where we have 60 percent of the vehicles going to the east and the rest 40 percent going to the west. In that case, we have:

$$\begin{array}{c} \rho_{e}=\frac{0.3*\frac{3}{3}*0.6}{1-0.6}=0.45 \end{array}$$

$$\begin{array}{c} \rho_{w}=\frac{0.3*\frac{3}{3}*\left(1-0.6\right)}{0.6}=0.2 \end{array}$$

Thus, we can estimate the corresponding flow with the fundamental diagram we obtained in our previous simulations (See Fig 1) using the new density values we have calculated. Hence, we have that for *ρ*_*e*_ and *ρ*_*w*_: The corresponding flow values are *q*_*e*_ = 0.21 and *q*_*w*_ = 0.29. To estimate the mean flow on each lane for this 3-3 lane configuration, we can do it in the following way:

$$\begin{array}{c} q_{3}-3=\frac{q_{e}*l_{e}+q_{w}*l_{w}}{l_{e}+l_{w}}=\frac{0.21*3+0.29*3}{3+3}=0.25 \end{array}$$

Using these equations, we can obtain the flow for any density and percentage number of vehicles to east direction value for any lane configuration. In the results section, we present the flow-density values we obtained for a six-lane scenario with reversible lanes. Using these values, we determine the configuration that maximizes traffic flow depending on the number of vehicles in each direction.

### Traffic data

To test our model, we use traffic data from the Transport Infrastructure Ireland https://www.tii.ie. They provide traffic volume information since 2006. The traffic counts data comes from induction loops embedded in the road surface. When a vehicle goes over one of these loops, a sensor detects it. We analyze the data to determine the traffic volume behavior during rush hours. We consider 852 datasets; each dataset contains the information from one sensor in the period comprehended from 2006 to 2015. We analyze more than five million records.

With the traffic data, we model three different scenarios. The first scenario is the non-reversible lane scenario, where we always have the same number of lanes for each direction. The second one is the reversible lane scenario, where we have a reversible lane in a specific direction over a fixed time interval. The third one is the adaptive lane scenario, where we have an adaptive lane that can change its direction depending on the traffic volume each hour.

For the non-reversible scenario, we assume a six-lane road with three lanes each side,i.e., a 3-3 lane configuration. So, we assign a 3-3 lane configuration to each record on the dataset. We obtain the average flow for that configuration from our model using the traffic volumes for each hour in every dataset to calculate the percentage of vehicles going to the east.

For the reversible scenario, we also assume a six-lane road. We calculate the average traffic volumes for the morning rush hour (from 6:00 am to 9:00 am). If the average traffic volume in the east direction is more than 50%, we assign a 2-4 lane configuration for all records in the morning rush hour; otherwise, we assign a 4-2 lane configuration. We obtain the mean flow for 2-4 and 4-2 configurations from our model using the traffic volumes at each record. We use the traffic volumes at each record in the morning rush hour from 6:00 am to 9:00 am for the adaptive scenario. For each record, we assign a lane configuration based on the results obtained our model on Fig 5. If the traffic volume to the east is between 0% and 41%, we assign the 4-2 configuration. If the east’s traffic volume is between 42% and 57%, we assign the 3-3 configuration. Otherwise, we assign the 2-4 configuration. As in the previous scenarios, we obtain the mean traffic flow for the corresponding configuration and traffic volume using the values obtained in our model. See Table 1 in the S1 File.

In the results section, we present a comparison between the three scenarios. We compare them in terms of traffic flow.

## Results

### Six-lane road with reversible lanes

**Fig 3:**
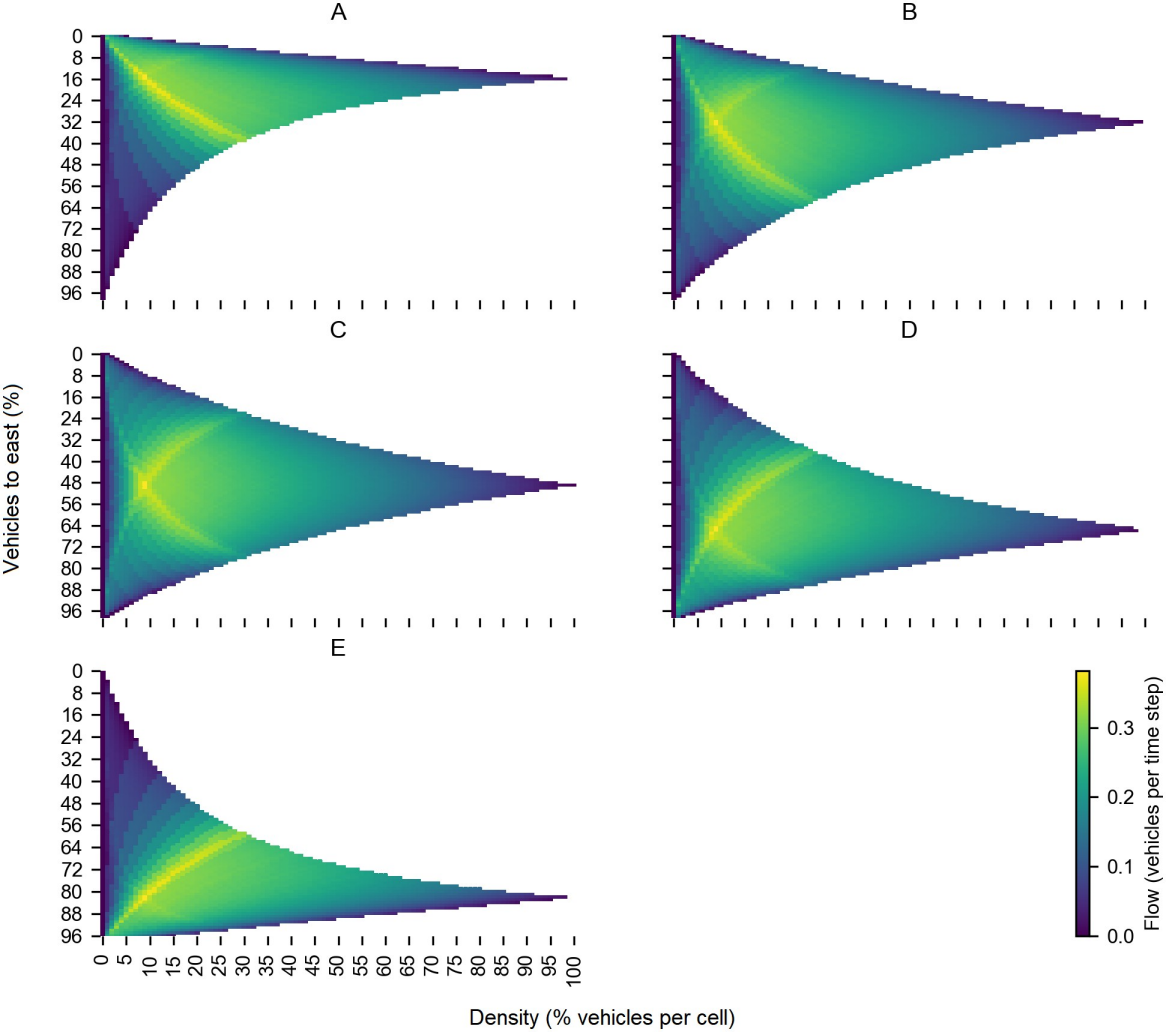
Heatmap of the traffic flow for all the lane configurations. We present the traffic flow in terms of vehicles’ density and the percentage of vehicles to the east. Higher values of traffic flow correspond to the bright yellow color. We observe how the maximum traffic flow peak changes accordingly with the lane configuration. (A)5-1 lane configuration. (B)4-2 lane configuration, (C)3-3 lane configuration. (D)2-4 lane configuration. (E)1-5 lane configuration.

We use the method described in the Reversible Lane Model section to model a six-lane road with reversible lanes. In Fig 3(A), we can observe the heatmap for the 5-1 lane configuration. We observe that the maximum flow values are in densities around 16%, and when the percentage of vehicles going to the east is around 20%. This behavior is because when we only have one lane to the east direction, the flow decreases if we add more than 20% vehicles to that direction due to the lower capacity of the road in the east direction. If the percentage of vehicles going to the east is around 20%, the flow will reach its maximum values because we maintain the optimal density values.

In Fig 3(C), we can see the heatmap for the 3-3 configuration. In this case, we can find higher flow values when around 50% of vehicles go to the east. This percentage corresponds to the symmetric scenario when the number of vehicles in each direction is the same; in this case the 3-3 configuration is optimal. We can also notice how flow decreases when the percentage of vehicles goes far higher or lower from 50%. We observe a similar behavior for 2-4, 4-2, and 1-5 lane configurations. In each heatmap, the optimal values of traffic flow change depending on which configuration we observe. With this information, we can see how traffic flow behaves concerning density and percentage of vehicles to the east, that is, the comportment of traffic flow for the different lane configurations.

**Fig 4:**
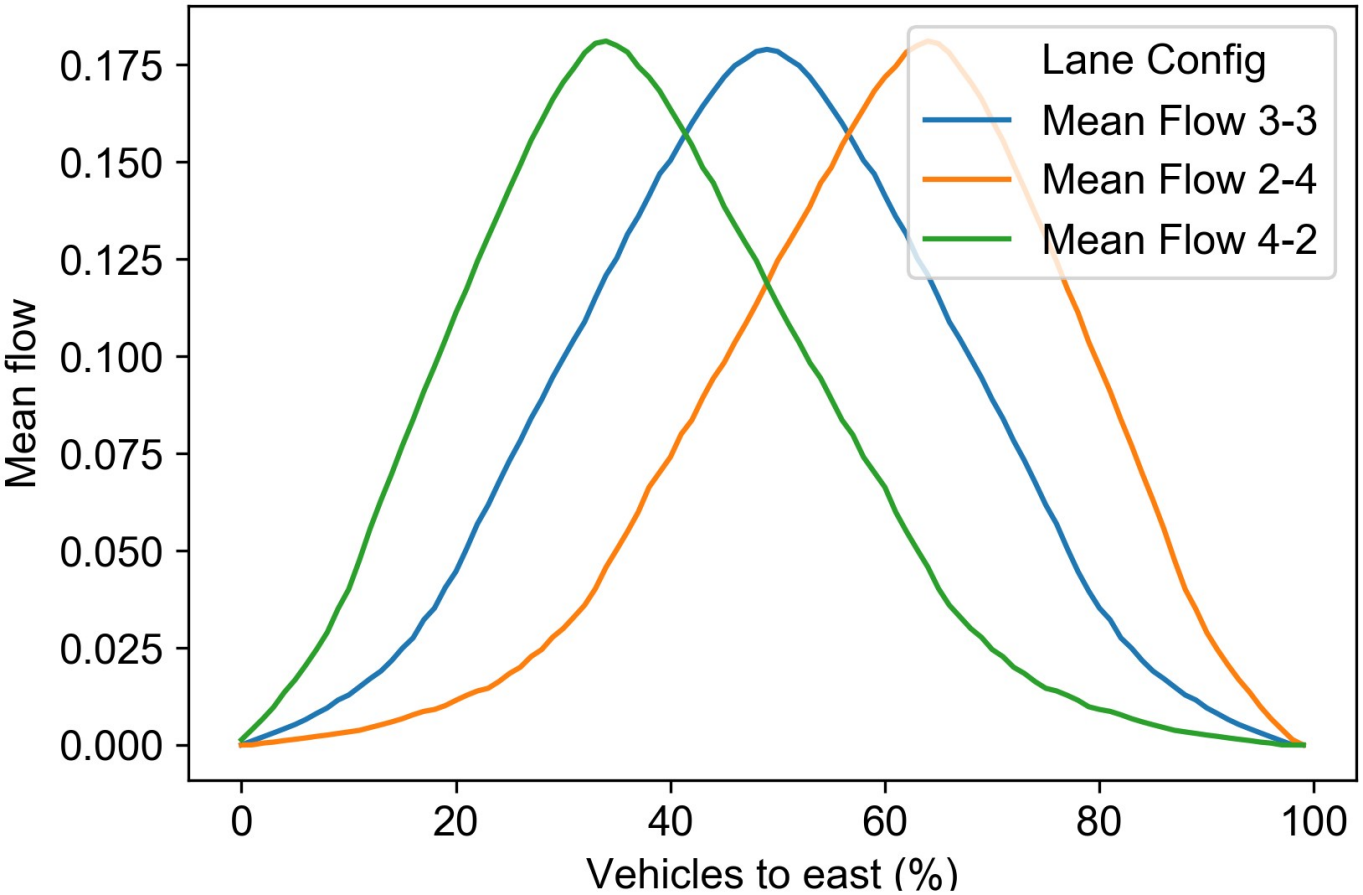
Mean flow by lane configuration. Mean flow as a function of the percentage of vehicles to the east direction for each lane configuration.

As a result, we can show that to maximize traffic flow, we only need to know the percentage of vehicles in each direction at a given moment and select the proper lane configuration. In Fig 4 for each lane configuration, we show the mean flow as a function of the east percentage. We can observe that for east percentage values from 0% to 41%, the lane configuration that maximizes flow is the 4-2, for 41% to 57% is the 3-3 lane configuration, and for 57% to 100% is the 2-4 lane configuration.

### Analysis of traffic asymmetry

**Fig 5:**
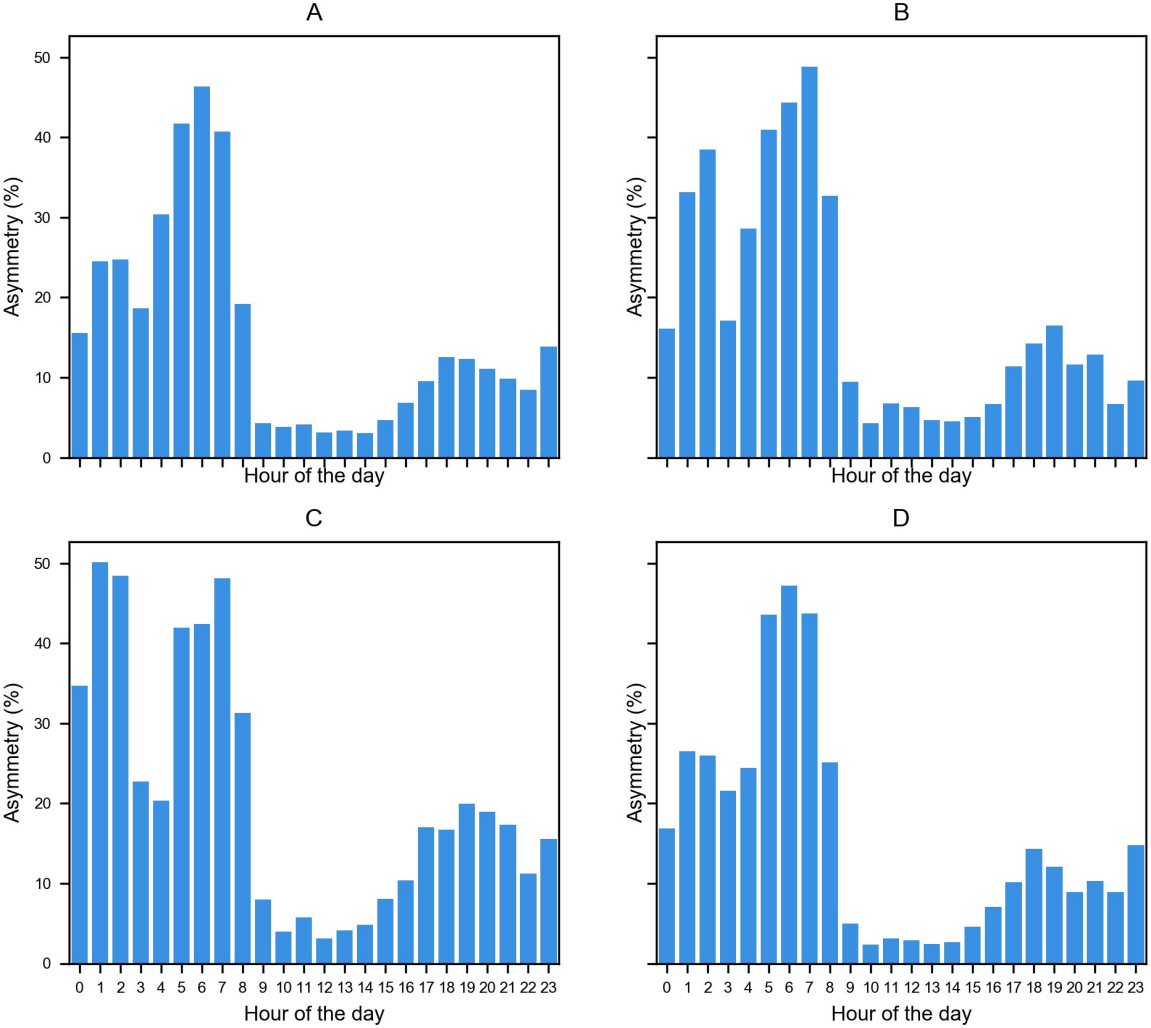
Distribution of asymmetry values of traffic volumes for a loop detector located in Dundalk. (A)Distribution of average asymmetry during 2013. (B)Distribution of average asymmetry in July 2013. (C)Distribution of average asymmetry between 15 and 19 of July 2013. (D)Distribution of asymmetry on July 17, 2013.

The datasets’ information described in the traffic data subsection allows us to calculate the asymmetry in the traffic volume for each loop detector. We define the asymmetry as 100*|*v*_*e*_ − *v*_*w*_|/*v*_*t*_. Where *v*_*e*_ is the east traffic volume, *v*_*w*_ is the west traffic volume and *v*_*t*_ is the total volume (*v*_*e*_ + *v*_*w*_). Thus, an asymmetry value of 0 indicates that *v*_*e*_ = *v*_*w*_ and a value of 100 is absolute asymmetry, *i.e*., either *v*_*e*_ = 0 or *v*_*w*_ = 0. In Fig 5, we observe the distribution of the asymmetry values for a sensor located in Dundalk at different time scales(year, month, week, and day). We observe a variance in the traffic asymmetry. This variance is important because, in the implementation of reversible lanes, it is prevalent that traffic planners use surveys to estimate traffic volume [35]. They use this method due to the costs of installing loop detectors, especially in developing countries; thus, the collected information via surveys could have a huge bias depending on the day and hour they acquire the information.

**Fig 6:**
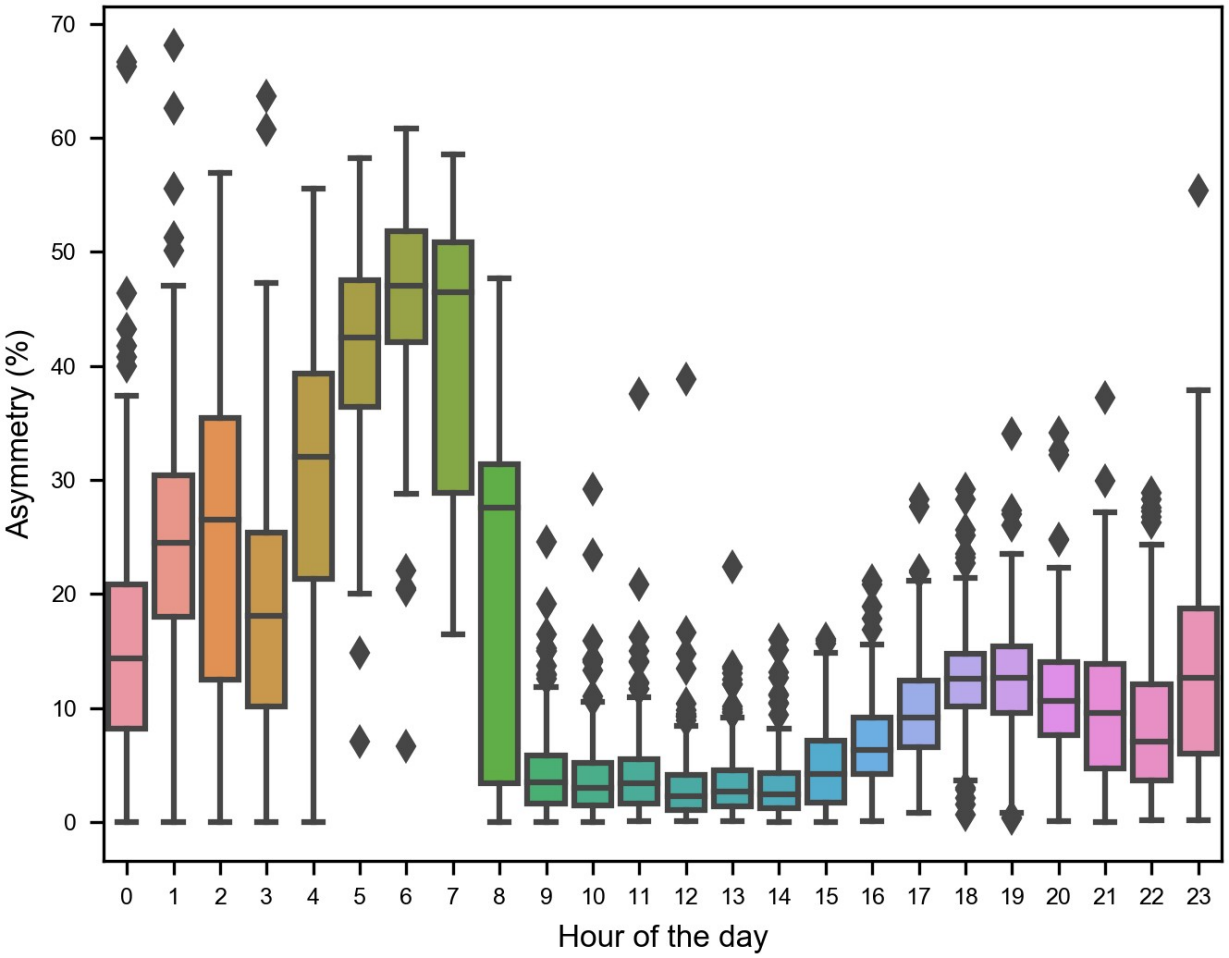
Boxplot of the traffic asymmetry for a loop detector located in Dundalk in 2013. We observe a high variance in the traffic volume during morning rush hours.

Even if traffic planners have loop detectors or other mechanisms to estimate traffic volume, our analysis shows that traffic volumes can have a high variance. In Fig 6, we present a boxplot to show the variance of the traffic asymmetry. Thus, it becomes tough to forecast traffic volume based on historical records or surveys. If our predicted traffic volumes are not accurate, the reversible lanes are not going to operate efficiently. Suppose the traffic volume increases in the opposite direction of the reversible lane. In that case, it could cause a bottleneck.This encourages us to expect that an adaptive reversible lane will perform better than a conventional reversible lane.

### Comparison between non-reversible, reversible and adaptive reversible scenarios

We modeled three scenarios using the historical traffic data https://www.tii.ie. A non-reversible scenario, a reversible scenario, and an adaptive reversible scenario. With our model, we obtained the mean traffic flow for each scenario for each of the 200 sensors during the morning rush hours (6 a.m. to 9 a.m).

**Fig 7:**
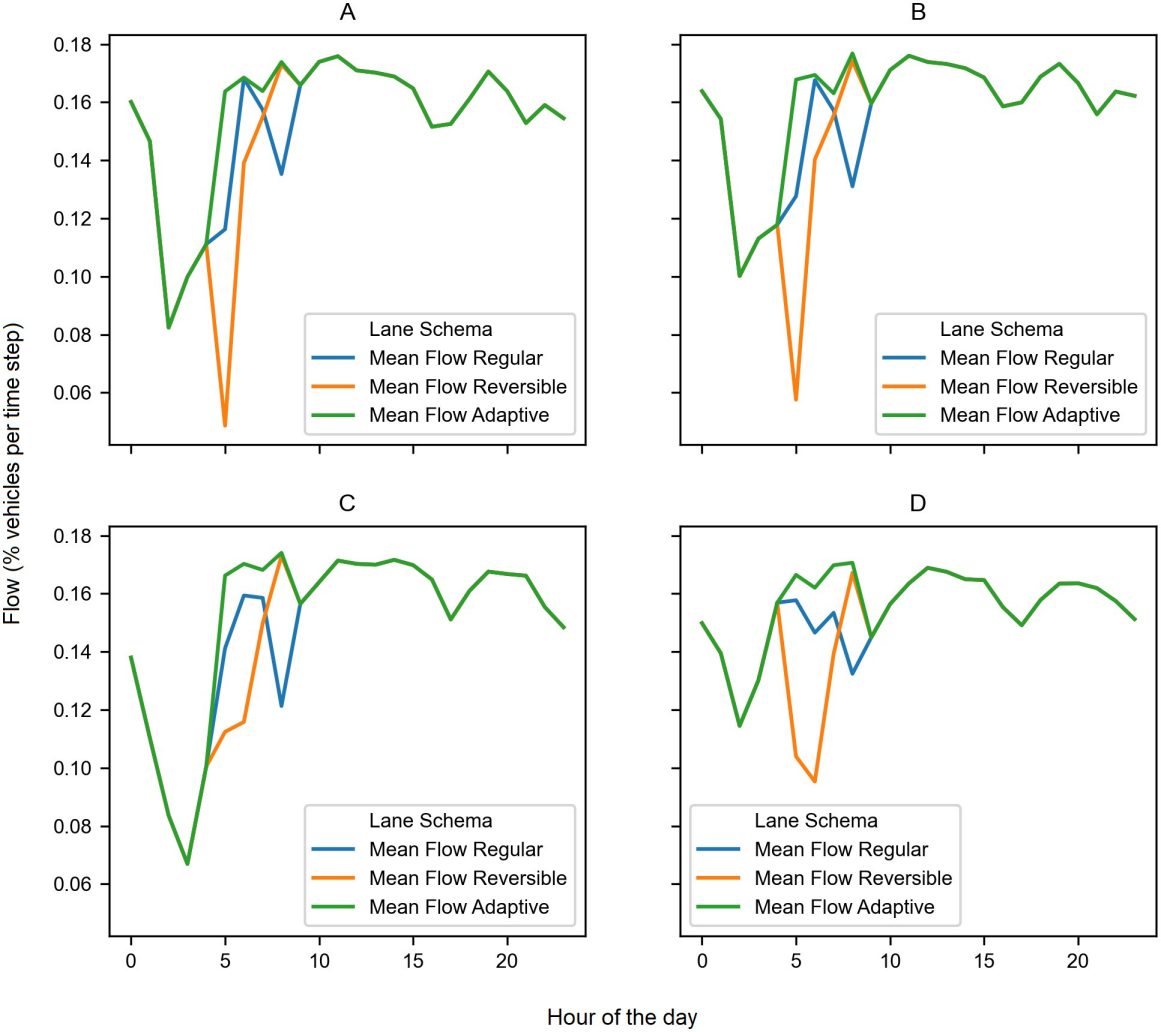
Mean flow for the three scenarios. In this figure, we show the mean flow for each scenario for the four locations. The adaptive lane scenario outperforms the reversible scenario. (A)Raheen 2012. (B)Rossbrien 2011. (C)Dunkettle 2007. (D)Rossbrien 2012.

For the sensors we selected, we observe that the mean flow is higher in the adaptive scenario than in the reversible scenario (see Fig 7). The fluctuation during the morning rush hours of the traffic volumes explains this increment in the traffic flow.

In the adaptive scenario, we have the option to change the configuration each hour from record to record; then, if the traffic volume changes significantly, the road can adapt to the current traffic volume with the proper lane configuration. On the other hand, in the reversible scenario, we maintain the same lane configuration during the morning rush hours, which is how conventional reversible lanes operate. Consequently, if the traffic volume changes significantly, the traffic flow decreases because that lane configuration is no longer optimal for the new traffic conditions.

We can also observe interesting comportment. In some cases, the non-reversible scenario performs better than the reversible scenario. When this occurs is because the traffic volumes fluctuate broadly.

### Configuration change ratio

We showed that the adaptive scenario augments traffic flow concerning the reversible scenario when traffic volume fluctuations occurs during the morning rush hours. Hence, we present a simple approach to characterize sensors by its traffic fluctuations.

When we modeled the three scenarios, we associated each record to a lane configuration depending on the scenario. Suppose the lane configurations in both scenarios are the same for a given record. In that case, that means there is no significant change in the traffic volume. Because if the traffic volume were significantly higher or lower than the rush hour’s average traffic volume, then we have assigned that record a different lane configuration for the adaptive scenario. Thereby, suppose the lane configurations are different for the reversible and the adaptive scenario. In that case, the traffic volume at that record is significantly higher or lower than the rush hour’s average traffic volume. In the first case, there is no change in the lane configuration in both scenarios; there is a change in the lane configuration in the second case. We can then calculate the configuration change ratio for each record on the dataset by counting the number of records that do not have the same lane configuration divided by the total number of records.

**Fig 8:**
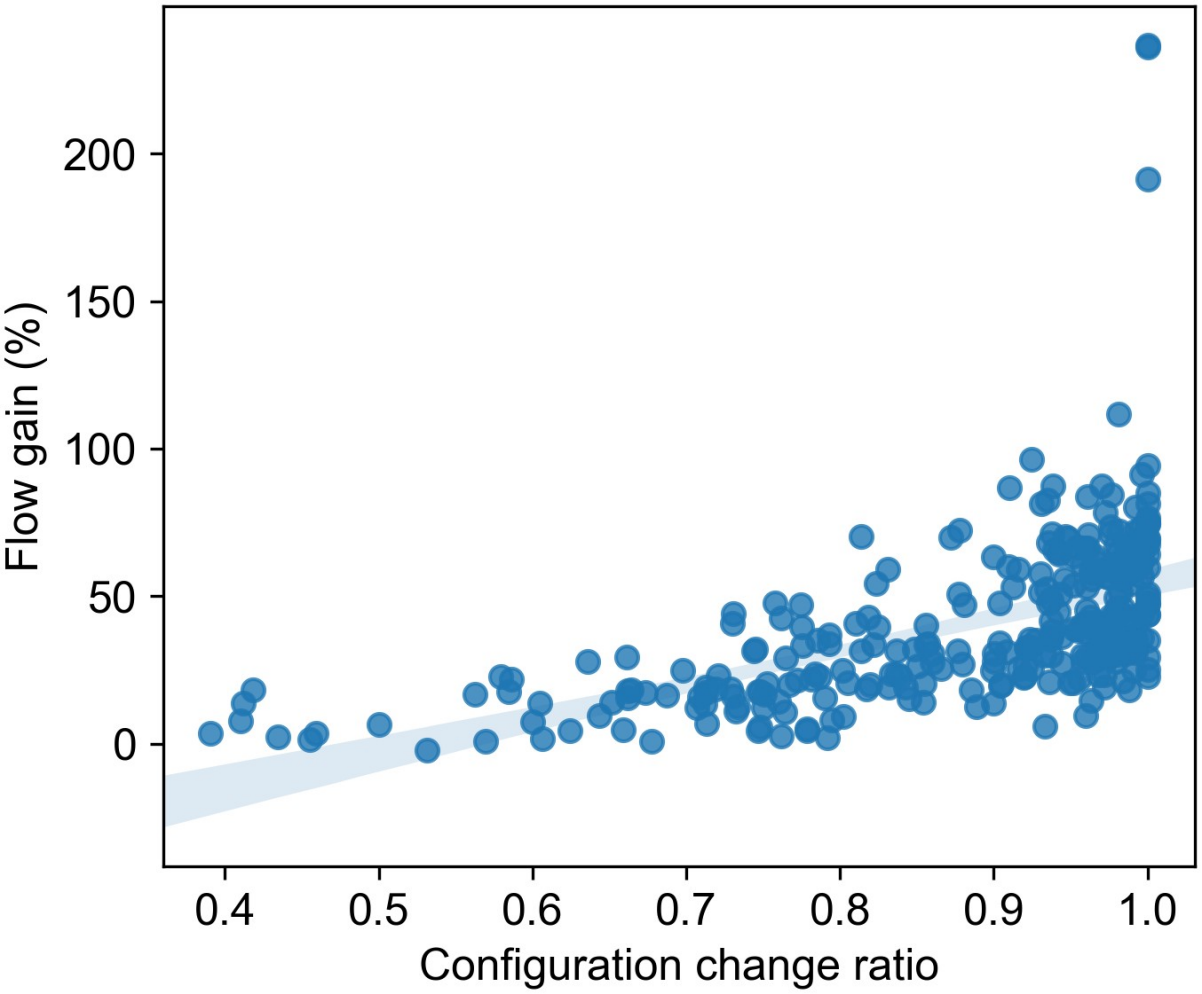
Configuration change ratio and traffic flow gain for the top 100 sensors. We show the flow gain for the top 100 sensors, which have a higher configuration change ratio. We can see a relation between higher values of flow gain and configuration change ratio.

We can use the configuration change ratio to determine which loop detectors (locations) could benefit more from the adaptive lanes. In Fig 8, we take the top 100 sensors ranked by its configuration change ratio. We observe a high correlation between the configuration change ratio and traffic flow gain(the flow difference between the reversible and the adaptive scenario). Thus, the more frequent the fluctuations in the traffic volume, the likely the adaptive reversible lane perform better than the conventional reversible lane. For the top 100 loop detectors, we have, on average, 40% more traffic flow for the adaptive scenario with respect to the reversible scenario.

## Discussion and conclusions

We presented a cellular automata approach to model adaptive reversible lanes. Our framework allows us to determine the potential benefits of adaptive reversible lanes based on historical traffic data. Unlike conventional reversible lanes, adaptive reversible lanes use data to adjust the lane’s direction on demand, depending on the traffic conditions. Reversible lanes take advantage of the asymmetry on traffic volumes during the rush hours; a pattern observed in most cities [6]. However, we have shown that traffic volume fluctuates wildly, even during rush hours. Because of this, adaptive lanes could perform better than conventional reversible lanes. The precise advantage of adaptive reversible lanes will depend on the asymmetry and variability of traffic and the mechanisms to implement them, varying from city to city. In any case, adaptive reversible lanes, in theory, may perform better than predefined reversible or regular lanes.

Limitations of our study include the modeling of lane changes [36–38], traffic intersections [17], and different driving behaviors [39]. Still, in previous work, we have considered each of these sophistications, so it would be straightforward to include them in future work and evaluate whether these features might affect the results presented in this work.

Authorities spend vast amounts of money on new vehicle infrastructure. However, a fraction of this infrastructure, specifically roads and highways, are underused because of the asymmetries on travel demand in modern cities. Adaptive lanes can optimize the use of the existing vehicle infrastructure by increasing the road capacity when needed. By doing that, authorities could save millions of dollars and invest more capital in public transport infrastructure and alternative mobility solutions, also distributing public space in a more equitable fashion.

One of the challenges of adaptive reversible lane implementation is the design of control systems. In [40], the authors propose two strategies to control the operation of a dynamical reversible lane. The control strategies they proposed based their functioning on the length of the bottleneck queue created by the reversible lane. Similar strategies could be developed using the number of vehicles as a parameter, as shown in this work. In [41], the authors propose a dynamic control to operate reversible lanes on signalized intersections. We could explore these ideas in the future using a more sophisticated cellular automata model.

However, we expect the full potential of adaptive lanes is in a self-driving car environment. In that sense, the implementation strategies should focus on the coordination between vehicles and infrastructure. Moreover, it could be interesting to estimate the time required for traffic to stabilize after lane changes take place under various conditions, which we leave for future work. Another essential feature of adaptive reversible lanes could be the possibility of handling different transport modes. This should be considered in future work. Other adaptation mechanisms could be integrated into the adaptive reversible lanes.

Another challenge is the physical mechanisms to operate the adaptive lanes safely. Transport engineers have designed strategies to make the implementation of conventional reversible lanes safer and more efficient. In [30], the authors present an automatic mechanism to separate the lanes physically without human intervention. In [31], authors propose a mechanism to control reversible lanes using real-time information. Safety impacts of roadway design have been studied in [26–29]. Transport engineers could develop other novel strategies to implement adaptive lanes. Assuming that it is impossible to implement adaptive lanes with the current technology, it is not senseless to assume that it might happen soon. We have been experiencing considerable progress in the autonomous vehicle industry, and it could be easier to implement adaptive lanes in a self-driving cars environment. We could dispense with centralized control for the adaptive lanes and take advantage of connected autonomous vehicles. The risk of accidents will be minimal after removing the human factor from the equation.

## Supporting information

S1 File (PDF)

S2 File (BIB)
