# Supplementary material for: Modeling adaptive reversible lanes: A cellular automata approach
Source: PLoS One. 2021 Jan 4;16(1):e0244326. doi: 10.1371/journal.pone.0244326 (PMC7781372; doi:10.1371/journal.pone.0244326)
Supplement: S1 File — (PDF) [file pone.0244326.s001.pdf]

Table 1: Example of a single processed file. Data from 2008 of a sensor located in Dunmore (N77-2).

Date	Vol North	Vol South	Asymmetry	South	Reversible	Adaptive	Regular	Flow Reversible	Flow Adaptive	Flow Regular
2008-01-01 05:00:00	20	12	25.0	37.5	4-2	2-4	3-3	0.05	0.18	0.13
2008-01-01 06:00:00	15	5	50.0	25.0	4-2	2-4	3-3	0.02	0.14	0.07
2008-01-01 07:00:00	16	30	30.43	65.22	4-2	4-2	3-3	0.18	0.18	0.12
2008-01-01 08:00:00	26	32	10.34	55.17	4-2	3-3	3-3	0.14	0.17	0.17
2008-01-02 05:00:00	36	26	16.13	41.94	4-2	3-3	3-3	0.07	0.15	0.15
2008-01-02 06:00:00	65	60	4.0	48.0	4-2	3-3	3-3	0.11	0.18	0.18
2008-01-02 07:00:00	147	182	10.64	55.32	4-2	3-3	3-3	0.14	0.17	0.17
2008-01-02 08:00:00	220	371	25.55	62.77	4-2	4-2	3-3	0.17	0.17	0.14
2008-01-03 05:00:00	26	22	8.33	45.83	4-2	3-3	3-3	0.09	0.17	0.17
2008-01-03 06:00:00	78	81	1.89	50.94	4-2	3-3	3-3	0.12	0.18	0.18
2008-01-03 07:00:00	234	306	13.33	56.67	4-2	3-3	3-3	0.15	0.16	0.16
2008-01-03 08:00:00	289	452	22.0	61.0	4-2	4-2	3-3	0.17	0.17	0.15
2008-01-04 05:00:00	34	28	9.68	45.16	4-2	3-3	3-3	0.09	0.17	0.17
2008-01-04 06:00:00	85	87	1.16	50.58	4-2	3-3	3-3	0.12	0.18	0.18
2008-01-04 07:00:00	218	293	14.68	57.34	4-2	4-2	3-3	0.15	0.15	0.16
2008-01-04 08:00:00	253	447	27.71	63.86	4-2	4-2	3-3	0.18	0.18	0.13
2008-01-07 05:00:00	52	26	33.33	33.33	4-2	2-4	3-3	0.04	0.18	0.11
2008-01-07 06:00:00	118	97	9.77	45.12	4-2	3-3	3-3	0.09	0.17	0.17
2008-01-07 07:00:00	320	361	6.02	53.01	4-2	3-3	3-3	0.13	0.17	0.17
2008-01-07 08:00:00	399	597	19.88	59.94	4-2	4-2	3-3	0.16	0.16	0.15
2008-01-08 05:00:00	37	24	21.31	39.34	4-2	2-4	3-3	0.07	0.17	0.14
2008-01-08 06:00:00	109	73	19.78	40.11	4-2	2-4	3-3	0.07	0.17	0.15
2008-01-08 07:00:00	295	383	12.98	56.49	4-2	3-3	3-3	0.15	0.16	0.16
2008-01-08 08:00:00	405	558	15.89	57.94	4-2	4-2	3-3	0.15	0.15	0.16
2008-01-09 05:00:00	39	21	30.0	35.0	4-2	2-4	3-3	0.05	0.18	0.12
2008-01-09 06:00:00	92	76	9.52	45.24	4-2	3-3	3-3	0.09	0.17	0.17
2008-01-09 07:00:00	286	336	8.04	54.02	4-2	3-3	3-3	0.14	0.17	0.17
2008-01-09 08:00:00	415	651	22.14	61.07	4-2	4-2	3-3	0.17	0.17	0.14
2008-01-10 05:00:00	38	20	31.03	34.48	4-2	2-4	3-3	0.04	0.18	0.12
2008-01-10 06:00:00	98	80	10.11	44.94	4-2	3-3	3-3	0.09	0.16	0.16
2008-01-10 07:00:00	283	349	10.44	55.22	4-2	3-3	3-3	0.14	0.17	0.17
2008-01-10 08:00:00	434	658	20.51	60.26	4-2	4-2	3-3	0.17	0.17	0.15
2008-01-11 05:00:00	33	19	26.92	36.54	4-2	2-4	3-3	0.05	0.18	0.13
2008-01-11 06:00:00	72	84	7.69	53.85	4-2	3-3	3-3	0.13	0.17	0.17
2008-01-11 07:00:00	259	357	15.91	57.95	4-2	4-2	3-3	0.15	0.15	0.16
2008-01-11 08:00:00	390	601	21.29	60.65	4-2	4-2	3-3	0.17	0.17	0.15
2008-01-14 05:00:00	54	26	35.0	32.5	4-2	2-4	3-3	0.03	0.17	0.1
2008-01-14 06:00:00	125	102	10.13	44.93	4-2	3-3	3-3	0.09	0.16	0.16
2008-01-14 07:00:00	313	385	10.32	55.16	4-2	3-3	3-3	0.14	0.17	0.17
2008-01-14 08:00:00	444	638	17.93	58.96	4-2	4-2	3-3	0.16	0.16	0.16
2008-01-15 05:00:00	37	24	21.31	39.34	4-2	2-4	3-3	0.07	0.17	0.14

Table 1: Example of a single processed file. Data from 2008 of a sensor located in Dunmore (N77-2).

Date	Vol North	Vol South	Asymmetry	South	Reversible	Adaptive	Regular	Flow Reversible	Flow Adaptive	Flow Regular
2008-01-15 06:00:00	108	89	9.64	45.18	4-2	3-3	3-3	0.09	0.17	0.17
2008-01-15 07:00:00	317	375	8.38	54.19	4-2	3-3	3-3	0.14	0.17	0.17
2008-01-15 08:00:00	445	640	17.97	58.99	4-2	4-2	3-3	0.16	0.16	0.16
2008-01-16 05:00:00	51	32	22.89	38.55	4-2	2-4	3-3	0.06	0.17	0.14
2008-01-16 06:00:00	115	87	13.86	43.07	4-2	3-3	3-3	0.08	0.16	0.16
2008-01-16 07:00:00	349	368	2.65	51.32	4-2	3-3	3-3	0.12	0.18	0.18
2008-01-16 08:00:00	427	624	18.74	59.37	4-2	4-2	3-3	0.16	0.16	0.15
2008-01-17 05:00:00	30	27	5.26	47.37	4-2	3-3	3-3	0.1	0.17	0.17
2008-01-17 06:00:00	112	79	17.28	41.36	4-2	3-3	3-3	0.07	0.15	0.15
2008-01-17 07:00:00	311	380	9.99	54.99	4-2	3-3	3-3	0.14	0.17	0.17
2008-01-17 08:00:00	448	602	14.67	57.33	4-2	4-2	3-3	0.15	0.15	0.16
2008-01-18 05:00:00	36	38	2.7	51.35	4-2	3-3	3-3	0.12	0.18	0.18
2008-01-18 06:00:00	107	77	16.3	41.85	4-2	3-3	3-3	0.07	0.15	0.15
2008-01-18 07:00:00	283	356	11.42	55.71	4-2	3-3	3-3	0.14	0.17	0.17
2008-01-18 08:00:00	446	591	13.98	56.99	4-2	3-3	3-3	0.15	0.16	0.16
2008-01-21 05:00:00	49	23	36.11	31.94	4-2	2-4	3-3	0.03	0.17	0.1
2008-01-21 06:00:00	128	104	10.34	44.83	4-2	3-3	3-3	0.09	0.16	0.16
2008-01-21 07:00:00	326	358	4.68	52.34	4-2	3-3	3-3	0.13	0.18	0.18
2008-01-21 08:00:00	436	644	19.26	59.63	4-2	4-2	3-3	0.16	0.16	0.15
2008-01-22 05:00:00	32	32	0.0	50.0	4-2	3-3	3-3	0.12	0.18	0.18
2008-01-22 06:00:00	118	89	14.01	43.0	4-2	3-3	3-3	0.08	0.16	0.16
2008-01-22 07:00:00	312	376	9.3	54.65	4-2	3-3	3-3	0.14	0.17	0.17
2008-01-22 08:00:00	456	623	15.48	57.74	4-2	4-2	3-3	0.15	0.15	0.16
2008-01-23 05:00:00	42	30	16.67	41.67	4-2	3-3	3-3	0.07	0.15	0.15
2008-01-23 06:00:00	109	79	15.96	42.02	4-2	3-3	3-3	0.08	0.16	0.16
2008-01-23 07:00:00	320	371	7.38	53.69	4-2	3-3	3-3	0.13	0.17	0.17
2008-01-23 08:00:00	442	626	17.23	58.61	4-2	4-2	3-3	0.16	0.16	0.16
2008-01-24 05:00:00	47	27	27.03	36.49	4-2	2-4	3-3	0.05	0.18	0.13
2008-01-24 06:00:00	125	94	14.16	42.92	4-2	3-3	3-3	0.08	0.16	0.16
2008-01-24 07:00:00	299	371	10.75	55.37	4-2	3-3	3-3	0.14	0.17	0.17
2008-01-24 08:00:00	459	642	16.62	58.31	4-2	4-2	3-3	0.16	0.16	0.16
2008-01-25 05:00:00	36	24	20.0	40.0	4-2	2-4	3-3	0.07	0.17	0.15
2008-01-25 06:00:00	91	78	7.69	46.15	4-2	3-3	3-3	0.1	0.17	0.17
2008-01-25 07:00:00	289	349	9.4	54.7	4-2	3-3	3-3	0.14	0.17	0.17
2008-01-25 08:00:00	426	545	12.26	56.13	4-2	3-3	3-3	0.15	0.16	0.16
2008-01-28 05:00:00	44	21	35.38	32.31	4-2	2-4	3-3	0.03	0.17	0.1
2008-01-28 06:00:00	116	96	9.43	45.28	4-2	3-3	3-3	0.09	0.17	0.17
2008-01-28 07:00:00	336	402	8.94	54.47	4-2	3-3	3-3	0.14	0.17	0.17
2008-01-28 08:00:00	444	652	18.98	59.49	4-2	4-2	3-3	0.16	0.16	0.15
2008-01-29 05:00:00	38	23	24.59	37.7	4-2	2-4	3-3	0.05	0.18	0.13
2008-01-29 06:00:00	101	92	4.66	47.67	4-2	3-3	3-3	0.1	0.17	0.17

Table 1: Example of a single processed file. Data from 2008 of a sensor located in Dunmore (N77-2).

Date	Vol North	Vol South	Asymmetry	South	Reversible	Adaptive	Regular	Flow Reversible	Flow Adaptive	Flow Regular
2008-01-29 07:00:00	344	377	4.58	52.29	4-2	3-3	3-3	0.13	0.18	0.18
2008-01-29 08:00:00	433	622	17.91	58.96	4-2	4-2	3-3	0.16	0.16	0.16
2008-01-30 05:00:00	38	31	10.14	44.93	4-2	3-3	3-3	0.09	0.16	0.16
2008-01-30 06:00:00	96	92	2.13	48.94	4-2	3-3	3-3	0.11	0.18	0.18
2008-01-30 07:00:00	326	369	6.19	53.09	4-2	3-3	3-3	0.13	0.17	0.17
2008-01-30 08:00:00	445	632	17.36	58.68	4-2	4-2	3-3	0.16	0.16	0.16
2008-01-31 05:00:00	44	29	20.55	39.73	4-2	2-4	3-3	0.07	0.17	0.14
2008-01-31 06:00:00	111	101	4.72	47.64	4-2	3-3	3-3	0.1	0.17	0.17
2008-01-31 07:00:00	313	395	11.58	55.79	4-2	3-3	3-3	0.14	0.17	0.17
2008-01-31 08:00:00	467	646	16.08	58.04	4-2	4-2	3-3	0.16	0.16	0.16
2008-02-01 05:00:00	47	39	9.3	45.35	4-2	3-3	3-3	0.09	0.17	0.17
2008-02-01 06:00:00	101	90	5.76	47.12	4-2	3-3	3-3	0.1	0.17	0.17
2008-02-01 07:00:00	262	350	14.38	57.19	4-2	4-2	3-3	0.15	0.15	0.16
2008-02-01 08:00:00	423	610	18.1	59.05	4-2	4-2	3-3	0.16	0.16	0.15
2008-02-04 05:00:00	50	22	38.89	30.56	4-2	2-4	3-3	0.03	0.17	0.09
2008-02-04 06:00:00	111	96	7.25	46.38	4-2	3-3	3-3	0.1	0.17	0.17
2008-02-04 07:00:00	317	355	5.65	52.83	4-2	3-3	3-3	0.13	0.18	0.18
2008-02-04 08:00:00	411	649	22.45	61.23	4-2	4-2	3-3	0.17	0.17	0.14
2008-02-05 05:00:00	31	23	14.81	42.59	4-2	3-3	3-3	0.08	0.16	0.16
2008-02-05 06:00:00	117	103	6.36	46.82	4-2	3-3	3-3	0.1	0.17	0.17
2008-02-05 07:00:00	324	364	5.81	52.91	4-2	3-3	3-3	0.13	0.18	0.18
2008-02-05 08:00:00	453	633	16.57	58.29	4-2	4-2	3-3	0.16	0.16	0.16
2008-02-06 05:00:00	40	24	25.0	37.5	4-2	2-4	3-3	0.05	0.18	0.13
2008-02-06 06:00:00	98	92	3.16	48.42	4-2	3-3	3-3	0.11	0.18	0.18
2008-02-06 07:00:00	342	333	1.33	49.33	4-2	3-3	3-3	0.11	0.18	0.18
2008-02-06 08:00:00	434	631	18.5	59.25	4-2	4-2	3-3	0.16	0.16	0.15
2008-02-07 05:00:00	48	33	18.52	40.74	4-2	2-4	3-3	0.07	0.17	0.15
2008-02-07 06:00:00	116	95	9.95	45.02	4-2	3-3	3-3	0.09	0.17	0.17
2008-02-07 07:00:00	312	360	7.14	53.57	4-2	3-3	3-3	0.13	0.17	0.17
2008-02-07 08:00:00	431	627	18.53	59.26	4-2	4-2	3-3	0.16	0.16	0.15
2008-02-08 05:00:00	43	20	36.51	31.75	4-2	2-4	3-3	0.03	0.17	0.1
2008-02-08 06:00:00	109	97	5.83	47.09	4-2	3-3	3-3	0.1	0.17	0.17
2008-02-08 07:00:00	300	345	6.98	53.49	4-2	3-3	3-3	0.13	0.17	0.17
2008-02-08 08:00:00	398	576	18.28	59.14	4-2	4-2	3-3	0.16	0.16	0.15
2008-02-11 05:00:00	58	27	36.47	31.76	4-2	2-4	3-3	0.03	0.17	0.1
2008-02-11 06:00:00	114	99	7.04	46.48	4-2	3-3	3-3	0.1	0.17	0.17
2008-02-11 07:00:00	325	343	2.69	51.35	4-2	3-3	3-3	0.12	0.18	0.18
2008-02-11 08:00:00	382	613	23.22	61.61	4-2	4-2	3-3	0.17	0.17	0.14
2008-02-12 05:00:00	31	26	8.77	45.61	4-2	3-3	3-3	0.09	0.17	0.17
2008-02-12 06:00:00	121	79	21.0	39.5	4-2	2-4	3-3	0.07	0.17	0.14
2008-02-12 07:00:00	305	366	9.09	54.55	4-2	3-3	3-3	0.14	0.17	0.17

Table 1: Example of a single processed file. Data from 2008 of a sensor located in Dunmore (N77-2).

Date	Vol North	Vol South	Asymmetry	South	Reversible	Adaptive	Regular	Flow Reversible	Flow Adaptive	Flow Regular
2008-02-12 08:00:00	430	605	16.91	58.45	4-2	4-2	3-3	0.16	0.16	0.16
2008-02-13 05:00:00	43	34	11.69	44.16	4-2	3-3	3-3	0.09	0.16	0.16
2008-02-13 06:00:00	104	104	0.0	50.0	4-2	3-3	3-3	0.12	0.18	0.18
2008-02-13 07:00:00	322	364	6.12	53.06	4-2	3-3	3-3	0.13	0.17	0.17
2008-02-13 08:00:00	388	593	20.9	60.45	4-2	4-2	3-3	0.17	0.17	0.15
2008-02-14 05:00:00	36	33	4.35	47.83	4-2	3-3	3-3	0.1	0.17	0.17
2008-02-14 06:00:00	118	105	5.83	47.09	4-2	3-3	3-3	0.1	0.17	0.17
2008-02-14 07:00:00	287	369	12.5	56.25	4-2	3-3	3-3	0.15	0.16	0.16
2008-02-14 08:00:00	360	570	22.58	61.29	4-2	4-2	3-3	0.17	0.17	0.14
2008-02-15 05:00:00	47	27	27.03	36.49	4-2	2-4	3-3	0.05	0.18	0.13
2008-02-15 06:00:00	104	85	10.05	44.97	4-2	3-3	3-3	0.09	0.16	0.16
2008-02-15 07:00:00	316	348	4.82	52.41	4-2	3-3	3-3	0.13	0.18	0.18
2008-02-15 08:00:00	329	539	24.19	62.1	4-2	4-2	3-3	0.17	0.17	0.14
2008-02-18 05:00:00	48	25	31.51	34.25	4-2	2-4	3-3	0.04	0.18	0.12
2008-02-18 06:00:00	116	99	7.91	46.05	4-2	3-3	3-3	0.1	0.17	0.17
2008-02-18 07:00:00	329	352	3.38	51.69	4-2	3-3	3-3	0.12	0.18	0.18
2008-02-18 08:00:00	425	632	19.58	59.79	4-2	4-2	3-3	0.16	0.16	0.15
2008-02-19 05:00:00	28	27	1.82	49.09	4-2	3-3	3-3	0.11	0.18	0.18
2008-02-19 06:00:00	112	91	10.34	44.83	4-2	3-3	3-3	0.09	0.16	0.16
2008-02-19 07:00:00	330	368	5.44	52.72	4-2	3-3	3-3	0.13	0.18	0.18
2008-02-19 08:00:00	437	609	16.44	58.22	4-2	4-2	3-3	0.16	0.16	0.16
2008-02-20 05:00:00	40	32	11.11	44.44	4-2	3-3	3-3	0.09	0.16	0.16
2008-02-20 06:00:00	112	97	7.18	46.41	4-2	3-3	3-3	0.1	0.17	0.17
2008-02-20 07:00:00	342	379	5.13	52.57	4-2	3-3	3-3	0.13	0.18	0.18
2008-02-20 08:00:00	461	630	15.49	57.75	4-2	4-2	3-3	0.15	0.15	0.16
2008-02-21 05:00:00	31	25	10.71	44.64	4-2	3-3	3-3	0.09	0.16	0.16
2008-02-21 06:00:00	112	94	8.74	45.63	4-2	3-3	3-3	0.09	0.17	0.17
2008-02-21 07:00:00	320	387	9.48	54.74	4-2	3-3	3-3	0.14	0.17	0.17
2008-02-21 08:00:00	469	620	13.87	56.93	4-2	3-3	3-3	0.15	0.16	0.16
2008-02-22 05:00:00	39	33	8.33	45.83	4-2	3-3	3-3	0.09	0.17	0.17
2008-02-22 06:00:00	101	99	1.0	49.5	4-2	3-3	3-3	0.11	0.18	0.18
2008-02-22 07:00:00	335	371	5.1	52.55	4-2	3-3	3-3	0.13	0.18	0.18
2008-02-22 08:00:00	411	623	20.5	60.25	4-2	4-2	3-3	0.17	0.17	0.15
2008-02-25 05:00:00	52	31	25.3	37.35	4-2	2-4	3-3	0.05	0.18	0.13
2008-02-25 06:00:00	125	122	1.21	49.39	4-2	3-3	3-3	0.11	0.18	0.18
2008-02-25 07:00:00	322	401	10.93	55.46	4-2	3-3	3-3	0.14	0.17	0.17
2008-02-25 08:00:00	434	629	18.34	59.17	4-2	4-2	3-3	0.16	0.16	0.15
2008-02-26 05:00:00	56	29	31.76	34.12	4-2	2-4	3-3	0.04	0.18	0.12
2008-02-26 06:00:00	112	118	2.61	51.3	4-2	3-3	3-3	0.12	0.18	0.18
2008-02-26 07:00:00	319	396	10.77	55.38	4-2	3-3	3-3	0.14	0.17	0.17
2008-02-26 08:00:00	457	626	15.6	57.8	4-2	4-2	3-3	0.15	0.15	0.16

Table 1: Example of a single processed file. Data from 2008 of a sensor located in Dunmore (N77-2).

Date	Vol North	Vol South	Asymmetry	South	Reversible	Adaptive	Regular	Flow Reversible	Flow Adaptive	Flow Regular
2008-02-27 05:00:00	47	41	6.82	46.59	4-2	3-3	3-3	0.1	0.17	0.17
2008-02-27 06:00:00	134	111	9.39	45.31	4-2	3-3	3-3	0.09	0.17	0.17
2008-02-27 07:00:00	323	375	7.45	53.72	4-2	3-3	3-3	0.13	0.17	0.17
2008-02-27 08:00:00	437	640	18.85	59.42	4-2	4-2	3-3	0.16	0.16	0.15
2008-02-28 05:00:00	38	32	8.57	45.71	4-2	3-3	3-3	0.09	0.17	0.17
2008-02-28 06:00:00	107	108	0.47	50.23	4-2	3-3	3-3	0.12	0.18	0.18
2008-02-28 07:00:00	317	414	13.27	56.63	4-2	3-3	3-3	0.15	0.16	0.16
2008-02-28 08:00:00	421	656	21.82	60.91	4-2	4-2	3-3	0.17	0.17	0.15
2008-02-29 05:00:00	47	28	25.33	37.33	4-2	2-4	3-3	0.05	0.18	0.13
2008-02-29 06:00:00	108	99	4.35	47.83	4-2	3-3	3-3	0.1	0.17	0.17
2008-02-29 07:00:00	318	383	9.27	54.64	4-2	3-3	3-3	0.14	0.17	0.17
2008-02-29 08:00:00	412	590	17.76	58.88	4-2	4-2	3-3	0.16	0.16	0.16
2008-03-03 05:00:00	50	25	33.33	33.33	4-2	2-4	3-3	0.04	0.18	0.11
2008-03-03 06:00:00	116	122	2.52	51.26	4-2	3-3	3-3	0.12	0.18	0.18
2008-03-03 07:00:00	284	404	17.44	58.72	4-2	4-2	3-3	0.16	0.16	0.16
2008-03-03 08:00:00	426	627	19.09	59.54	4-2	4-2	3-3	0.16	0.16	0.15
2008-03-04 05:00:00	48	27	28.0	36.0	4-2	2-4	3-3	0.05	0.18	0.13
2008-03-04 06:00:00	107	102	2.39	48.8	4-2	3-3	3-3	0.11	0.18	0.18
2008-03-04 07:00:00	340	374	4.76	52.38	4-2	3-3	3-3	0.13	0.18	0.18
2008-03-04 08:00:00	456	657	18.06	59.03	4-2	4-2	3-3	0.16	0.16	0.15
2008-03-05 05:00:00	56	31	28.74	35.63	4-2	2-4	3-3	0.05	0.18	0.12
2008-03-05 06:00:00	114	113	0.44	49.78	4-2	3-3	3-3	0.11	0.18	0.18
2008-03-05 07:00:00	341	379	5.28	52.64	4-2	3-3	3-3	0.13	0.18	0.18
2008-03-05 08:00:00	425	632	19.58	59.79	4-2	4-2	3-3	0.16	0.16	0.15
2008-03-06 05:00:00	40	24	25.0	37.5	4-2	2-4	3-3	0.05	0.18	0.13
2008-03-06 06:00:00	132	111	8.64	45.68	4-2	3-3	3-3	0.09	0.17	0.17
2008-03-06 07:00:00	334	404	9.49	54.74	4-2	3-3	3-3	0.14	0.17	0.17
2008-03-06 08:00:00	478	636	14.18	57.09	4-2	4-2	3-3	0.15	0.15	0.16
2008-03-07 05:00:00	47	41	6.82	46.59	4-2	3-3	3-3	0.1	0.17	0.17
2008-03-07 06:00:00	106	105	0.47	49.76	4-2	3-3	3-3	0.11	0.18	0.18
2008-03-07 07:00:00	328	366	5.48	52.74	4-2	3-3	3-3	0.13	0.18	0.18
2008-03-07 08:00:00	442	612	16.13	58.06	4-2	4-2	3-3	0.16	0.16	0.16
2008-03-10 05:00:00	50	24	35.14	32.43	4-2	2-4	3-3	0.03	0.17	0.1
2008-03-10 06:00:00	125	106	8.23	45.89	4-2	3-3	3-3	0.09	0.17	0.17
2008-03-10 07:00:00	337	384	6.52	53.26	4-2	3-3	3-3	0.13	0.17	0.17
2008-03-10 08:00:00	408	672	24.44	62.22	4-2	4-2	3-3	0.17	0.17	0.14
2008-03-11 05:00:00	48	30	23.08	38.46	4-2	2-4	3-3	0.06	0.17	0.14
2008-03-11 06:00:00	124	117	2.9	48.55	4-2	3-3	3-3	0.11	0.18	0.18
2008-03-11 07:00:00	355	391	4.83	52.41	4-2	3-3	3-3	0.13	0.18	0.18
2008-03-11 08:00:00	476	631	14.0	57.0	4-2	4-2	3-3	0.15	0.15	0.16
2008-03-12 05:00:00	50	27	29.87	35.06	4-2	2-4	3-3	0.05	0.18	0.12

Table 1: Example of a single processed file. Data from 2008 of a sensor located in Dunmore (N77-2).

Date	Vol North	Vol South	Asymmetry	South	Reversible	Adaptive	Regular	Flow Reversible	Flow Adaptive	Flow Regular
2008-03-12 06:00:00	106	118	5.36	52.68	4-2	3-3	3-3	0.13	0.18	0.18
2008-03-12 07:00:00	344	389	6.14	53.07	4-2	3-3	3-3	0.13	0.17	0.17
2008-03-12 08:00:00	429	626	18.67	59.34	4-2	4-2	3-3	0.16	0.16	0.15
2008-03-13 05:00:00	52	36	18.18	40.91	4-2	2-4	3-3	0.07	0.17	0.15
2008-03-13 06:00:00	136	136	0.0	50.0	4-2	3-3	3-3	0.12	0.18	0.18
2008-03-13 07:00:00	342	404	8.31	54.16	4-2	3-3	3-3	0.14	0.17	0.17
2008-03-13 08:00:00	444	609	15.67	57.83	4-2	4-2	3-3	0.15	0.15	0.16
2008-03-14 05:00:00	47	35	14.63	42.68	4-2	3-3	3-3	0.08	0.16	0.16
2008-03-14 06:00:00	119	101	8.18	45.91	4-2	3-3	3-3	0.09	0.17	0.17
2008-03-14 07:00:00	294	370	11.45	55.72	4-2	3-3	3-3	0.14	0.17	0.17
2008-03-14 08:00:00	371	604	23.9	61.95	4-2	4-2	3-3	0.17	0.17	0.14
2008-03-17 05:00:00	11	11	0.0	50.0	4-2	3-3	3-3	0.12	0.18	0.18
2008-03-17 06:00:00	23	17	15.0	42.5	4-2	3-3	3-3	0.08	0.16	0.16
2008-03-17 07:00:00	51	62	9.73	54.87	4-2	3-3	3-3	0.14	0.17	0.17
2008-03-17 08:00:00	98	85	7.1	46.45	4-2	3-3	3-3	0.1	0.17	0.17
2008-03-18 05:00:00	43	28	21.13	39.44	4-2	2-4	3-3	0.07	0.17	0.14
2008-03-18 06:00:00	133	105	11.76	44.12	4-2	3-3	3-3	0.09	0.16	0.16
2008-03-18 07:00:00	299	360	9.26	54.63	4-2	3-3	3-3	0.14	0.17	0.17
2008-03-18 08:00:00	368	555	20.26	60.13	4-2	4-2	3-3	0.17	0.17	0.15
2008-03-19 05:00:00	42	28	20.0	40.0	4-2	2-4	3-3	0.07	0.17	0.15
2008-03-19 06:00:00	110	115	2.22	51.11	4-2	3-3	3-3	0.12	0.18	0.18
2008-03-19 07:00:00	306	392	12.32	56.16	4-2	3-3	3-3	0.15	0.16	0.16
2008-03-19 08:00:00	370	573	21.53	60.76	4-2	4-2	3-3	0.17	0.17	0.15
2008-03-20 05:00:00	45	30	20.0	40.0	4-2	2-4	3-3	0.07	0.17	0.15
2008-03-20 06:00:00	127	121	2.42	48.79	4-2	3-3	3-3	0.11	0.18	0.18
2008-03-20 07:00:00	291	423	18.49	59.24	4-2	4-2	3-3	0.16	0.16	0.15
2008-03-20 08:00:00	413	530	12.41	56.2	4-2	3-3	3-3	0.15	0.16	0.16
2008-03-21 05:00:00	16	33	34.69	67.35	4-2	4-2	3-3	0.18	0.18	0.11
2008-03-21 06:00:00	92	68	15.0	42.5	4-2	3-3	3-3	0.08	0.16	0.16
2008-03-21 07:00:00	181	197	4.23	52.12	4-2	3-3	3-3	0.13	0.18	0.18
2008-03-21 08:00:00	271	251	3.83	48.08	4-2	3-3	3-3	0.11	0.18	0.18
2008-03-24 05:00:00	8	11	15.79	57.89	4-2	4-2	3-3	0.15	0.15	0.16
2008-03-24 06:00:00	23	9	43.75	28.12	4-2	2-4	3-3	0.02	0.16	0.08
2008-03-24 07:00:00	54	65	9.24	54.62	4-2	3-3	3-3	0.14	0.17	0.17
2008-03-24 08:00:00	78	80	1.27	50.63	4-2	3-3	3-3	0.12	0.18	0.18
2008-03-25 05:00:00	47	31	20.51	39.74	4-2	2-4	3-3	0.07	0.17	0.14
2008-03-25 06:00:00	115	87	13.86	43.07	4-2	3-3	3-3	0.08	0.16	0.16
2008-03-25 07:00:00	300	337	5.81	52.9	4-2	3-3	3-3	0.13	0.18	0.18
2008-03-25 08:00:00	358	586	24.15	62.08	4-2	4-2	3-3	0.17	0.17	0.14
2008-03-26 05:00:00	40	38	2.56	48.72	4-2	3-3	3-3	0.11	0.18	0.18
2008-03-26 06:00:00	119	100	8.68	45.66	4-2	3-3	3-3	0.09	0.17	0.17

Table 1: Example of a single processed file. Data from 2008 of a sensor located in Dunmore (N77-2).

Date	Vol North	Vol South	Asymmetry	South	Reversible	Adaptive	Regular	Flow Reversible	Flow Adaptive	Flow Regular
2008-03-26 07:00:00	285	340	8.8	54.4	4-2	3-3	3-3	0.14	0.17	0.17
2008-03-26 08:00:00	363	584	23.34	61.67	4-2	4-2	3-3	0.17	0.17	0.14
2008-03-27 05:00:00	40	44	4.76	52.38	4-2	3-3	3-3	0.13	0.18	0.18
2008-03-27 06:00:00	134	100	14.53	42.74	4-2	3-3	3-3	0.08	0.16	0.16
2008-03-27 07:00:00	263	372	17.17	58.58	4-2	4-2	3-3	0.16	0.16	0.16
2008-03-27 08:00:00	366	568	21.63	60.81	4-2	4-2	3-3	0.17	0.17	0.15
2008-03-28 05:00:00	38	22	26.67	36.67	4-2	2-4	3-3	0.05	0.18	0.13
2008-03-28 06:00:00	113	97	7.62	46.19	4-2	3-3	3-3	0.1	0.17	0.17
2008-03-28 07:00:00	261	341	13.29	56.64	4-2	3-3	3-3	0.15	0.16	0.16
2008-03-28 08:00:00	340	548	23.42	61.71	4-2	4-2	3-3	0.17	0.17	0.14
2008-03-31 05:00:00	54	25	36.71	31.65	4-2	2-4	3-3	0.03	0.17	0.1
2008-03-31 06:00:00	131	101	12.93	43.53	4-2	3-3	3-3	0.08	0.16	0.16
2008-03-31 07:00:00	309	382	10.56	55.28	4-2	3-3	3-3	0.14	0.17	0.17
2008-03-31 08:00:00	422	667	22.5	61.25	4-2	4-2	3-3	0.17	0.17	0.14
2008-04-01 05:00:00	43	28	21.13	39.44	4-2	2-4	3-3	0.07	0.17	0.14
2008-04-01 06:00:00	118	87	15.12	42.44	4-2	3-3	3-3	0.08	0.16	0.16
2008-04-01 07:00:00	317	407	12.43	56.22	4-2	3-3	3-3	0.15	0.16	0.16
2008-04-01 08:00:00	430	678	22.38	61.19	4-2	4-2	3-3	0.17	0.17	0.14
2008-04-02 05:00:00	50	31	23.46	38.27	4-2	2-4	3-3	0.06	0.17	0.14
2008-04-02 06:00:00	118	101	7.76	46.12	4-2	3-3	3-3	0.1	0.17	0.17
2008-04-02 07:00:00	341	400	7.96	53.98	4-2	3-3	3-3	0.13	0.17	0.17
2008-04-02 08:00:00	440	644	18.82	59.41	4-2	4-2	3-3	0.16	0.16	0.15
2008-04-03 05:00:00	48	34	17.07	41.46	4-2	3-3	3-3	0.07	0.15	0.15
2008-04-03 06:00:00	115	118	1.29	50.64	4-2	3-3	3-3	0.12	0.18	0.18
2008-04-03 07:00:00	331	417	11.5	55.75	4-2	3-3	3-3	0.14	0.17	0.17
2008-04-03 08:00:00	451	668	19.39	59.7	4-2	4-2	3-3	0.16	0.16	0.15
2008-04-04 05:00:00	48	25	31.51	34.25	4-2	2-4	3-3	0.04	0.18	0.12
2008-04-04 06:00:00	123	109	6.03	46.98	4-2	3-3	3-3	0.1	0.17	0.17
2008-04-04 07:00:00	297	408	15.74	57.87	4-2	4-2	3-3	0.15	0.15	0.16
2008-04-04 08:00:00	402	679	25.62	62.81	4-2	4-2	3-3	0.17	0.17	0.14
2008-04-07 05:00:00	46	26	27.78	36.11	4-2	2-4	3-3	0.05	0.18	0.13
2008-04-07 06:00:00	112	105	3.23	48.39	4-2	3-3	3-3	0.11	0.18	0.18
2008-04-07 07:00:00	337	420	10.96	55.48	4-2	3-3	3-3	0.14	0.17	0.17
2008-04-07 08:00:00	402	634	22.39	61.2	4-2	4-2	3-3	0.17	0.17	0.14
2008-04-08 05:00:00	38	30	11.76	44.12	4-2	3-3	3-3	0.09	0.16	0.16
2008-04-08 06:00:00	127	105	9.48	45.26	4-2	3-3	3-3	0.09	0.17	0.17
2008-04-08 07:00:00	301	384	12.12	56.06	4-2	3-3	3-3	0.15	0.16	0.16
2008-04-08 08:00:00	457	663	18.39	59.2	4-2	4-2	3-3	0.16	0.16	0.15
2008-04-09 05:00:00	58	31	30.34	34.83	4-2	2-4	3-3	0.04	0.18	0.12
2008-04-09 06:00:00	124	107	7.36	46.32	4-2	3-3	3-3	0.1	0.17	0.17
2008-04-09 07:00:00	349	415	8.64	54.32	4-2	3-3	3-3	0.14	0.17	0.17

Table 1: Example of a single processed file. Data from 2008 of a sensor located in Dunmore (N77-2).

Date	Vol North	Vol South	Asymmetry	South	Reversible	Adaptive	Regular	Flow Reversible	Flow Adaptive	Flow Regular
2008-04-09 08:00:00	418	622	19.62	59.81	4-2	4-2	3-3	0.16	0.16	0.15
2008-04-10 05:00:00	48	31	21.52	39.24	4-2	2-4	3-3	0.07	0.17	0.14
2008-04-10 06:00:00	126	136	3.82	51.91	4-2	3-3	3-3	0.12	0.18	0.18
2008-04-10 07:00:00	330	404	10.08	55.04	4-2	3-3	3-3	0.14	0.17	0.17
2008-04-10 08:00:00	455	639	16.82	58.41	4-2	4-2	3-3	0.16	0.16	0.16
2008-04-11 05:00:00	40	35	6.67	46.67	4-2	3-3	3-3	0.1	0.17	0.17
2008-04-11 06:00:00	104	109	2.35	51.17	4-2	3-3	3-3	0.12	0.18	0.18
2008-04-11 07:00:00	322	382	8.52	54.26	4-2	3-3	3-3	0.14	0.17	0.17
2008-04-11 08:00:00	426	613	18.0	59.0	4-2	4-2	3-3	0.16	0.16	0.16
2008-04-14 05:00:00	52	25	35.06	32.47	4-2	2-4	3-3	0.03	0.17	0.1
2008-04-14 06:00:00	131	117	5.65	47.18	4-2	3-3	3-3	0.1	0.17	0.17
2008-04-14 07:00:00	368	412	5.64	52.82	4-2	3-3	3-3	0.13	0.18	0.18
2008-04-14 08:00:00	446	692	21.62	60.81	4-2	4-2	3-3	0.17	0.17	0.15
2008-04-15 05:00:00	47	30	22.08	38.96	4-2	2-4	3-3	0.06	0.17	0.14
2008-04-15 06:00:00	126	127	0.4	50.2	4-2	3-3	3-3	0.12	0.18	0.18
2008-04-15 07:00:00	339	400	8.25	54.13	4-2	3-3	3-3	0.14	0.17	0.17
2008-04-15 08:00:00	471	652	16.12	58.06	4-2	4-2	3-3	0.16	0.16	0.16
2008-04-16 05:00:00	44	27	23.94	38.03	4-2	2-4	3-3	0.06	0.17	0.14
2008-04-16 06:00:00	151	114	13.96	43.02	4-2	3-3	3-3	0.08	0.16	0.16
2008-04-16 07:00:00	343	403	8.04	54.02	4-2	3-3	3-3	0.14	0.17	0.17
2008-04-16 08:00:00	430	667	21.6	60.8	4-2	4-2	3-3	0.17	0.17	0.15
2008-04-17 05:00:00	44	32	15.79	42.11	4-2	3-3	3-3	0.08	0.16	0.16
2008-04-17 06:00:00	138	127	4.15	47.92	4-2	3-3	3-3	0.1	0.17	0.17
2008-04-17 07:00:00	339	413	9.84	54.92	4-2	3-3	3-3	0.14	0.17	0.17
2008-04-17 08:00:00	452	658	18.56	59.28	4-2	4-2	3-3	0.16	0.16	0.15
2008-04-18 05:00:00	46	28	24.32	37.84	4-2	2-4	3-3	0.05	0.18	0.13
2008-04-18 06:00:00	132	121	4.35	47.83	4-2	3-3	3-3	0.1	0.17	0.17
2008-04-18 07:00:00	312	363	7.56	53.78	4-2	3-3	3-3	0.13	0.17	0.17
2008-04-18 08:00:00	373	618	24.72	62.36	4-2	4-2	3-3	0.17	0.17	0.14
2008-04-21 05:00:00	60	30	33.33	33.33	4-2	2-4	3-3	0.04	0.18	0.11
2008-04-21 06:00:00	128	120	3.23	48.39	4-2	3-3	3-3	0.11	0.18	0.18
2008-04-21 07:00:00	344	419	9.83	54.91	4-2	3-3	3-3	0.14	0.17	0.17
2008-04-21 08:00:00	431	666	21.42	60.71	4-2	4-2	3-3	0.17	0.17	0.15
2008-04-22 05:00:00	41	28	18.84	40.58	4-2	2-4	3-3	0.07	0.17	0.15
2008-04-22 06:00:00	115	116	0.43	50.22	4-2	3-3	3-3	0.12	0.18	0.18
2008-04-22 07:00:00	366	406	5.18	52.59	4-2	3-3	3-3	0.13	0.18	0.18
2008-04-22 08:00:00	447	638	17.6	58.8	4-2	4-2	3-3	0.16	0.16	0.16
2008-04-23 05:00:00	49	34	18.07	40.96	4-2	2-4	3-3	0.07	0.17	0.15
2008-04-23 06:00:00	124	122	0.81	49.59	4-2	3-3	3-3	0.11	0.18	0.18
2008-04-23 07:00:00	343	415	9.5	54.75	4-2	3-3	3-3	0.14	0.17	0.17
2008-04-23 08:00:00	460	647	16.89	58.45	4-2	4-2	3-3	0.16	0.16	0.16

Table 1: Example of a single processed file. Data from 2008 of a sensor located in Dunmore (N77-2).

Date	Vol North	Vol South	Asymmetry	South	Reversible	Adaptive	Regular	Flow Reversible	Flow Adaptive	Flow Regular
2008-04-24 05:00:00	40	43	3.61	51.81	4-2	3-3	3-3	0.12	0.18	0.18
2008-04-24 06:00:00	128	131	1.16	50.58	4-2	3-3	3-3	0.12	0.18	0.18
2008-04-24 07:00:00	338	398	8.15	54.08	4-2	3-3	3-3	0.14	0.17	0.17
2008-04-24 08:00:00	455	643	17.12	58.56	4-2	4-2	3-3	0.16	0.16	0.16
2008-04-25 05:00:00	46	27	26.03	36.99	4-2	2-4	3-3	0.05	0.18	0.13
2008-04-25 06:00:00	132	90	18.92	40.54	4-2	2-4	3-3	0.07	0.17	0.15
2008-04-25 07:00:00	306	386	11.56	55.78	4-2	3-3	3-3	0.14	0.17	0.17
2008-04-25 08:00:00	422	602	17.58	58.79	4-2	4-2	3-3	0.16	0.16	0.16
2008-04-28 05:00:00	71	29	42.0	29.0	4-2	2-4	3-3	0.02	0.16	0.09
2008-04-28 06:00:00	118	125	2.88	51.44	4-2	3-3	3-3	0.12	0.18	0.18
2008-04-28 07:00:00	336	411	10.04	55.02	4-2	3-3	3-3	0.14	0.17	0.17
2008-04-28 08:00:00	424	655	21.41	60.7	4-2	4-2	3-3	0.17	0.17	0.15
2008-04-29 05:00:00	37	24	21.31	39.34	4-2	2-4	3-3	0.07	0.17	0.14
2008-04-29 06:00:00	119	128	3.64	51.82	4-2	3-3	3-3	0.12	0.18	0.18
2008-04-29 07:00:00	368	429	7.65	53.83	4-2	3-3	3-3	0.13	0.17	0.17
2008-04-29 08:00:00	477	616	12.72	56.36	4-2	3-3	3-3	0.15	0.16	0.16
2008-04-30 05:00:00	55	30	29.41	35.29	4-2	2-4	3-3	0.05	0.18	0.12
2008-04-30 06:00:00	123	128	1.99	51.0	4-2	3-3	3-3	0.12	0.18	0.18
2008-04-30 07:00:00	349	421	9.35	54.68	4-2	3-3	3-3	0.14	0.17	0.17
2008-04-30 08:00:00	474	649	15.58	57.79	4-2	4-2	3-3	0.15	0.15	0.16
2008-05-01 05:00:00	52	45	7.22	46.39	4-2	3-3	3-3	0.1	0.17	0.17
2008-05-01 06:00:00	127	132	1.93	50.97	4-2	3-3	3-3	0.12	0.18	0.18
2008-05-01 07:00:00	366	417	6.51	53.26	4-2	3-3	3-3	0.13	0.17	0.17
2008-05-01 08:00:00	410	669	24.0	62.0	4-2	4-2	3-3	0.17	0.17	0.14
2008-05-02 05:00:00	36	29	10.77	44.62	4-2	3-3	3-3	0.09	0.16	0.16
2008-05-02 06:00:00	114	111	1.33	49.33	4-2	3-3	3-3	0.11	0.18	0.18
2008-05-02 07:00:00	305	372	9.9	54.95	4-2	3-3	3-3	0.14	0.17	0.17
2008-05-02 08:00:00	392	583	19.59	59.79	4-2	4-2	3-3	0.16	0.16	0.15
2008-05-05 05:00:00	16	13	10.34	44.83	4-2	3-3	3-3	0.09	0.16	0.16
2008-05-05 06:00:00	26	19	15.56	42.22	4-2	3-3	3-3	0.08	0.16	0.16
2008-05-05 07:00:00	60	75	11.11	55.56	4-2	3-3	3-3	0.14	0.17	0.17
2008-05-05 08:00:00	98	77	12.0	44.0	4-2	3-3	3-3	0.09	0.16	0.16
2008-05-06 05:00:00	63	37	26.0	37.0	4-2	2-4	3-3	0.05	0.18	0.13
2008-05-06 06:00:00	158	139	6.4	46.8	4-2	3-3	3-3	0.1	0.17	0.17
2008-05-06 07:00:00	325	425	13.33	56.67	4-2	3-3	3-3	0.15	0.16	0.16
2008-05-06 08:00:00	398	645	23.68	61.84	4-2	4-2	3-3	0.17	0.17	0.14
2008-05-07 05:00:00	55	34	23.6	38.2	4-2	2-4	3-3	0.06	0.17	0.14
2008-05-07 06:00:00	146	130	5.8	47.1	4-2	3-3	3-3	0.1	0.17	0.17
2008-05-07 07:00:00	329	408	10.72	55.36	4-2	3-3	3-3	0.14	0.17	0.17
2008-05-07 08:00:00	441	642	18.56	59.28	4-2	4-2	3-3	0.16	0.16	0.15
2008-05-08 05:00:00	46	43	3.37	48.31	4-2	3-3	3-3	0.11	0.18	0.18

Table 1: Example of a single processed file. Data from 2008 of a sensor located in Dunmore (N77-2).

Date	Vol North	Vol South	Asymmetry	South	Reversible	Adaptive	Regular	Flow Reversible	Flow Adaptive	Flow Regular
2008-05-08 06:00:00	133	134	0.37	50.19	4-2	3-3	3-3	0.12	0.18	0.18
2008-05-08 07:00:00	343	401	7.8	53.9	4-2	3-3	3-3	0.13	0.17	0.17
2008-05-08 08:00:00	433	641	19.37	59.68	4-2	4-2	3-3	0.16	0.16	0.15
2008-05-09 05:00:00	48	40	9.09	45.45	4-2	3-3	3-3	0.09	0.17	0.17
2008-05-09 06:00:00	128	133	1.92	50.96	4-2	3-3	3-3	0.12	0.18	0.18
2008-05-09 07:00:00	301	374	10.81	55.41	4-2	3-3	3-3	0.14	0.17	0.17
2008-05-09 08:00:00	443	648	18.79	59.4	4-2	4-2	3-3	0.16	0.16	0.15
2008-05-12 05:00:00	70	36	32.08	33.96	4-2	2-4	3-3	0.04	0.18	0.11
2008-05-12 06:00:00	132	150	6.38	53.19	4-2	3-3	3-3	0.13	0.17	0.17
2008-05-12 07:00:00	334	401	9.12	54.56	4-2	3-3	3-3	0.14	0.17	0.17
2008-05-12 08:00:00	456	630	16.02	58.01	4-2	4-2	3-3	0.16	0.16	0.16
2008-05-13 05:00:00	55	43	12.24	43.88	4-2	3-3	3-3	0.08	0.16	0.16
2008-05-13 06:00:00	129	136	2.64	51.32	4-2	3-3	3-3	0.12	0.18	0.18
2008-05-13 07:00:00	345	378	4.56	52.28	4-2	3-3	3-3	0.13	0.18	0.18
2008-05-13 08:00:00	503	662	13.65	56.82	4-2	3-3	3-3	0.15	0.16	0.16
2008-05-14 05:00:00	44	49	5.38	52.69	4-2	3-3	3-3	0.13	0.18	0.18
2008-05-14 06:00:00	151	119	11.85	44.07	4-2	3-3	3-3	0.09	0.16	0.16
2008-05-14 07:00:00	326	397	9.82	54.91	4-2	3-3	3-3	0.14	0.17	0.17
2008-05-14 08:00:00	468	683	18.68	59.34	4-2	4-2	3-3	0.16	0.16	0.15
2008-05-15 05:00:00	56	46	9.8	45.1	4-2	3-3	3-3	0.09	0.17	0.17
2008-05-15 06:00:00	139	151	4.14	52.07	4-2	3-3	3-3	0.13	0.18	0.18
2008-05-15 07:00:00	353	407	7.11	53.55	4-2	3-3	3-3	0.13	0.17	0.17
2008-05-15 08:00:00	464	708	20.82	60.41	4-2	4-2	3-3	0.17	0.17	0.15
2008-05-16 05:00:00	44	40	4.76	47.62	4-2	3-3	3-3	0.1	0.17	0.17
2008-05-16 06:00:00	135	121	5.47	47.27	4-2	3-3	3-3	0.1	0.17	0.17
2008-05-16 07:00:00	312	374	9.04	54.52	4-2	3-3	3-3	0.14	0.17	0.17
2008-05-16 08:00:00	435	618	17.38	58.69	4-2	4-2	3-3	0.16	0.16	0.16
2008-05-19 05:00:00	66	37	28.16	35.92	4-2	2-4	3-3	0.05	0.18	0.12
2008-05-19 06:00:00	129	137	3.01	51.5	4-2	3-3	3-3	0.12	0.18	0.18
2008-05-19 07:00:00	332	420	11.7	55.85	4-2	3-3	3-3	0.14	0.17	0.17
2008-05-19 08:00:00	434	695	23.12	61.56	4-2	4-2	3-3	0.17	0.17	0.14
2008-05-20 05:00:00	59	36	24.21	37.89	4-2	2-4	3-3	0.05	0.18	0.13
2008-05-20 06:00:00	122	139	6.51	53.26	4-2	3-3	3-3	0.13	0.17	0.17
2008-05-20 07:00:00	322	373	7.34	53.67	4-2	3-3	3-3	0.13	0.17	0.17
2008-05-20 08:00:00	445	668	20.04	60.02	4-2	4-2	3-3	0.17	0.17	0.15
2008-05-21 05:00:00	56	45	10.89	44.55	4-2	3-3	3-3	0.09	0.16	0.16
2008-05-21 06:00:00	142	130	4.41	47.79	4-2	3-3	3-3	0.1	0.17	0.17
2008-05-21 07:00:00	338	417	10.46	55.23	4-2	3-3	3-3	0.14	0.17	0.17
2008-05-21 08:00:00	474	616	13.03	56.51	4-2	3-3	3-3	0.15	0.16	0.16
2008-05-22 05:00:00	58	44	13.73	43.14	4-2	3-3	3-3	0.08	0.16	0.16
2008-05-22 06:00:00	130	130	0.0	50.0	4-2	3-3	3-3	0.12	0.18	0.18

Table 1: Example of a single processed file. Data from 2008 of a sensor located in Dunmore (N77-2).

Date	Vol North	Vol South	Asymmetry	South	Reversible	Adaptive	Regular	Flow Reversible	Flow Adaptive	Flow Regular
2008-05-22 07:00:00	317	414	13.27	56.63	4-2	3-3	3-3	0.15	0.16	0.16
2008-05-22 08:00:00	473	647	15.54	57.77	4-2	4-2	3-3	0.15	0.15	0.16
2008-05-23 05:00:00	55	36	20.88	39.56	4-2	2-4	3-3	0.07	0.17	0.14
2008-05-23 06:00:00	128	137	3.4	51.7	4-2	3-3	3-3	0.12	0.18	0.18
2008-05-23 07:00:00	322	371	7.07	53.54	4-2	3-3	3-3	0.13	0.17	0.17
2008-05-23 08:00:00	401	582	18.41	59.21	4-2	4-2	3-3	0.16	0.16	0.15
2008-05-26 05:00:00	50	29	26.58	36.71	4-2	2-4	3-3	0.05	0.18	0.13
2008-05-26 06:00:00	128	145	6.23	53.11	4-2	3-3	3-3	0.13	0.17	0.17
2008-05-26 07:00:00	347	431	10.8	55.4	4-2	3-3	3-3	0.14	0.17	0.17
2008-05-26 08:00:00	450	637	17.2	58.6	4-2	4-2	3-3	0.16	0.16	0.16
2008-05-27 05:00:00	49	31	22.5	38.75	4-2	2-4	3-3	0.06	0.17	0.14
2008-05-27 06:00:00	132	131	0.38	49.81	4-2	3-3	3-3	0.11	0.18	0.18
2008-05-27 07:00:00	338	404	8.89	54.45	4-2	3-3	3-3	0.14	0.17	0.17
2008-05-27 08:00:00	456	599	13.55	56.78	4-2	3-3	3-3	0.15	0.16	0.16
2008-05-28 05:00:00	54	31	27.06	36.47	4-2	2-4	3-3	0.05	0.18	0.13
2008-05-28 06:00:00	129	138	3.37	51.69	4-2	3-3	3-3	0.12	0.18	0.18
2008-05-28 07:00:00	349	403	7.18	53.59	4-2	3-3	3-3	0.13	0.17	0.17
2008-05-28 08:00:00	439	626	17.56	58.78	4-2	4-2	3-3	0.16	0.16	0.16
2008-05-29 05:00:00	55	44	11.11	44.44	4-2	3-3	3-3	0.09	0.16	0.16
2008-05-29 06:00:00	130	160	10.34	55.17	4-2	3-3	3-3	0.14	0.17	0.17
2008-05-29 07:00:00	362	411	6.34	53.17	4-2	3-3	3-3	0.13	0.17	0.17
2008-05-29 08:00:00	435	673	21.48	60.74	4-2	4-2	3-3	0.17	0.17	0.15
2008-05-30 05:00:00	45	39	7.14	46.43	4-2	3-3	3-3	0.1	0.17	0.17
2008-05-30 06:00:00	122	128	2.4	51.2	4-2	3-3	3-3	0.12	0.18	0.18
2008-05-30 07:00:00	263	360	15.57	57.78	4-2	4-2	3-3	0.15	0.15	0.16
2008-05-30 08:00:00	402	550	15.55	57.77	4-2	4-2	3-3	0.15	0.15	0.16
2008-06-02 05:00:00	20	11	29.03	35.48	4-2	2-4	3-3	0.05	0.18	0.12
2008-06-02 06:00:00	25	21	8.7	45.65	4-2	3-3	3-3	0.09	0.17	0.17
2008-06-02 07:00:00	49	66	14.78	57.39	4-2	4-2	3-3	0.15	0.15	0.16
2008-06-02 08:00:00	101	98	1.51	49.25	4-2	3-3	3-3	0.11	0.18	0.18
2008-06-03 05:00:00	59	36	24.21	37.89	4-2	2-4	3-3	0.05	0.18	0.13
2008-06-03 06:00:00	122	137	5.79	52.9	4-2	3-3	3-3	0.13	0.18	0.18
2008-06-03 07:00:00	351	396	6.02	53.01	4-2	3-3	3-3	0.13	0.17	0.17
2008-06-03 08:00:00	404	582	18.05	59.03	4-2	4-2	3-3	0.16	0.16	0.15
2008-06-04 05:00:00	50	29	26.58	36.71	4-2	2-4	3-3	0.05	0.18	0.13
2008-06-04 06:00:00	132	132	0.0	50.0	4-2	3-3	3-3	0.12	0.18	0.18
2008-06-04 07:00:00	343	393	6.79	53.4	4-2	3-3	3-3	0.13	0.17	0.17
2008-06-04 08:00:00	424	622	18.93	59.46	4-2	4-2	3-3	0.16	0.16	0.15
2008-06-05 05:00:00	41	37	5.13	47.44	4-2	3-3	3-3	0.1	0.17	0.17
2008-06-05 06:00:00	134	123	4.28	47.86	4-2	3-3	3-3	0.1	0.17	0.17
2008-06-05 07:00:00	345	414	9.09	54.55	4-2	3-3	3-3	0.14	0.17	0.17

Table 1: Example of a single processed file. Data from 2008 of a sensor located in Dunmore (N77-2).

Date	Vol North	Vol South	Asymmetry	South	Reversible	Adaptive	Regular	Flow Reversible	Flow Adaptive	Flow Regular
2008-06-05 08:00:00	395	637	23.45	61.72	4-2	4-2	3-3	0.17	0.17	0.14
2008-06-06 05:00:00	43	31	16.22	41.89	4-2	3-3	3-3	0.07	0.15	0.15
2008-06-06 06:00:00	103	137	14.17	57.08	4-2	4-2	3-3	0.15	0.15	0.16
2008-06-06 07:00:00	317	397	11.2	55.6	4-2	3-3	3-3	0.14	0.17	0.17
2008-06-06 08:00:00	384	595	21.55	60.78	4-2	4-2	3-3	0.17	0.17	0.15
2008-06-09 05:00:00	47	36	13.25	43.37	4-2	3-3	3-3	0.08	0.16	0.16
2008-06-09 06:00:00	133	118	5.98	47.01	4-2	3-3	3-3	0.1	0.17	0.17
2008-06-09 07:00:00	316	390	10.48	55.24	4-2	3-3	3-3	0.14	0.17	0.17
2008-06-09 08:00:00	403	615	20.83	60.41	4-2	4-2	3-3	0.17	0.17	0.15
2008-06-10 05:00:00	46	42	4.55	47.73	4-2	3-3	3-3	0.1	0.17	0.17
2008-06-10 06:00:00	120	139	7.34	53.67	4-2	3-3	3-3	0.13	0.17	0.17
2008-06-10 07:00:00	362	410	6.22	53.11	4-2	3-3	3-3	0.13	0.17	0.17
2008-06-10 08:00:00	432	652	20.3	60.15	4-2	4-2	3-3	0.17	0.17	0.15
2008-06-11 05:00:00	44	30	18.92	40.54	4-2	2-4	3-3	0.07	0.17	0.15
2008-06-11 06:00:00	128	136	3.03	51.52	4-2	3-3	3-3	0.12	0.18	0.18
2008-06-11 07:00:00	356	399	5.7	52.85	4-2	3-3	3-3	0.13	0.18	0.18
2008-06-11 08:00:00	399	613	21.15	60.57	4-2	4-2	3-3	0.17	0.17	0.15
2008-06-12 05:00:00	46	42	4.55	47.73	4-2	3-3	3-3	0.1	0.17	0.17
2008-06-12 06:00:00	127	148	7.64	53.82	4-2	3-3	3-3	0.13	0.17	0.17
2008-06-12 07:00:00	330	411	10.93	55.47	4-2	3-3	3-3	0.14	0.17	0.17
2008-06-12 08:00:00	386	601	21.78	60.89	4-2	4-2	3-3	0.17	0.17	0.15
2008-06-13 05:00:00	48	33	18.52	40.74	4-2	2-4	3-3	0.07	0.17	0.15
2008-06-13 06:00:00	117	125	3.31	51.65	4-2	3-3	3-3	0.12	0.18	0.18
2008-06-13 07:00:00	292	407	16.45	58.23	4-2	4-2	3-3	0.16	0.16	0.16
2008-06-13 08:00:00	371	562	20.47	60.24	4-2	4-2	3-3	0.17	0.17	0.15
2008-06-16 05:00:00	56	36	21.74	39.13	4-2	2-4	3-3	0.07	0.17	0.14
2008-06-16 06:00:00	130	141	4.06	52.03	4-2	3-3	3-3	0.13	0.18	0.18
2008-06-16 07:00:00	326	402	10.44	55.22	4-2	3-3	3-3	0.14	0.17	0.17
2008-06-16 08:00:00	395	612	21.55	60.77	4-2	4-2	3-3	0.17	0.17	0.15
2008-06-17 05:00:00	49	28	27.27	36.36	4-2	2-4	3-3	0.05	0.18	0.13
2008-06-17 06:00:00	121	131	3.97	51.98	4-2	3-3	3-3	0.12	0.18	0.18
2008-06-17 07:00:00	344	411	8.87	54.44	4-2	3-3	3-3	0.14	0.17	0.17
2008-06-17 08:00:00	425	628	19.28	59.64	4-2	4-2	3-3	0.16	0.16	0.15
2008-06-18 05:00:00	57	36	22.58	38.71	4-2	2-4	3-3	0.06	0.17	0.14
2008-06-18 06:00:00	111	146	13.62	56.81	4-2	3-3	3-3	0.15	0.16	0.16
2008-06-18 07:00:00	350	377	3.71	51.86	4-2	3-3	3-3	0.12	0.18	0.18
2008-06-18 08:00:00	426	624	18.86	59.43	4-2	4-2	3-3	0.16	0.16	0.15
2008-06-19 05:00:00	48	33	18.52	40.74	4-2	2-4	3-3	0.07	0.17	0.15
2008-06-19 06:00:00	128	157	10.18	55.09	4-2	3-3	3-3	0.14	0.17	0.17
2008-06-19 07:00:00	341	410	9.19	54.59	4-2	3-3	3-3	0.14	0.17	0.17
2008-06-19 08:00:00	420	654	21.79	60.89	4-2	4-2	3-3	0.17	0.17	0.15

Table 1: Example of a single processed file. Data from 2008 of a sensor located in Dunmore (N77-2).

Date	Vol North	Vol South	Asymmetry	South	Reversible	Adaptive	Regular	Flow Reversible	Flow Adaptive	Flow Regular
2008-06-20 05:00:00	47	27	27.03	36.49	4-2	2-4	3-3	0.05	0.18	0.13
2008-06-20 06:00:00	124	141	6.42	53.21	4-2	3-3	3-3	0.13	0.17	0.17
2008-06-20 07:00:00	299	383	12.32	56.16	4-2	3-3	3-3	0.15	0.16	0.16
2008-06-20 08:00:00	408	609	19.76	59.88	4-2	4-2	3-3	0.16	0.16	0.15
2008-06-23 05:00:00	54	51	2.86	48.57	4-2	3-3	3-3	0.11	0.18	0.18
2008-06-23 06:00:00	121	130	3.59	51.79	4-2	3-3	3-3	0.12	0.18	0.18
2008-06-23 07:00:00	332	409	10.39	55.2	4-2	3-3	3-3	0.14	0.17	0.17
2008-06-23 08:00:00	394	591	20.0	60.0	4-2	4-2	3-3	0.17	0.17	0.15
2008-06-24 05:00:00	38	28	15.15	42.42	4-2	3-3	3-3	0.08	0.16	0.16
2008-06-24 06:00:00	140	141	0.36	50.18	4-2	3-3	3-3	0.12	0.18	0.18
2008-06-24 07:00:00	311	398	12.27	56.14	4-2	3-3	3-3	0.15	0.16	0.16
2008-06-24 08:00:00	397	589	19.47	59.74	4-2	4-2	3-3	0.16	0.16	0.15
2008-06-25 05:00:00	43	32	14.67	42.67	4-2	3-3	3-3	0.08	0.16	0.16
2008-06-25 06:00:00	125	148	8.42	54.21	4-2	3-3	3-3	0.14	0.17	0.17
2008-06-25 07:00:00	315	373	8.43	54.22	4-2	3-3	3-3	0.14	0.17	0.17
2008-06-25 08:00:00	392	563	17.91	58.95	4-2	4-2	3-3	0.16	0.16	0.16
2008-06-26 05:00:00	41	31	13.89	43.06	4-2	3-3	3-3	0.08	0.16	0.16
2008-06-26 06:00:00	128	158	10.49	55.24	4-2	3-3	3-3	0.14	0.17	0.17
2008-06-26 07:00:00	313	403	12.57	56.28	4-2	3-3	3-3	0.15	0.16	0.16
2008-06-26 08:00:00	367	599	24.02	62.01	4-2	4-2	3-3	0.17	0.17	0.14
2008-06-27 05:00:00	42	43	1.18	50.59	4-2	3-3	3-3	0.12	0.18	0.18
2008-06-27 06:00:00	98	125	12.11	56.05	4-2	3-3	3-3	0.15	0.16	0.16
2008-06-27 07:00:00	304	370	9.79	54.9	4-2	3-3	3-3	0.14	0.17	0.17
2008-06-27 08:00:00	344	538	22.0	61.0	4-2	4-2	3-3	0.17	0.17	0.15
2008-06-30 05:00:00	61	39	22.0	39.0	4-2	2-4	3-3	0.07	0.17	0.14
2008-06-30 06:00:00	136	152	5.56	52.78	4-2	3-3	3-3	0.13	0.18	0.18
2008-06-30 07:00:00	335	412	10.31	55.15	4-2	3-3	3-3	0.14	0.17	0.17
2008-06-30 08:00:00	322	561	27.07	63.53	4-2	4-2	3-3	0.18	0.18	0.13
2008-07-01 05:00:00	43	36	8.86	45.57	4-2	3-3	3-3	0.09	0.17	0.17
2008-07-01 06:00:00	129	130	0.39	50.19	4-2	3-3	3-3	0.12	0.18	0.18
2008-07-01 07:00:00	319	368	7.13	53.57	4-2	3-3	3-3	0.13	0.17	0.17
2008-07-01 08:00:00	361	582	23.44	61.72	4-2	4-2	3-3	0.17	0.17	0.14
2008-07-02 05:00:00	51	41	10.87	44.57	4-2	3-3	3-3	0.09	0.16	0.16
2008-07-02 06:00:00	152	160	2.56	51.28	4-2	3-3	3-3	0.12	0.18	0.18
2008-07-02 07:00:00	365	566	21.59	60.79	4-2	4-2	3-3	0.17	0.17	0.15
2008-07-02 08:00:00	457	770	25.51	62.75	4-2	4-2	3-3	0.17	0.17	0.14
2008-07-03 05:00:00	55	50	4.76	47.62	4-2	3-3	3-3	0.1	0.17	0.17
2008-07-03 06:00:00	149	182	9.97	54.98	4-2	3-3	3-3	0.14	0.17	0.17
2008-07-03 07:00:00	407	581	17.61	58.81	4-2	4-2	3-3	0.16	0.16	0.16
2008-07-03 08:00:00	464	791	26.06	63.03	4-2	4-2	3-3	0.18	0.18	0.13
2008-07-04 05:00:00	49	49	0.0	50.0	4-2	3-3	3-3	0.12	0.18	0.18

Table 1: Example of a single processed file. Data from 2008 of a sensor located in Dunmore (N77-2).

Date	Vol North	Vol South	Asymmetry	South	Reversible	Adaptive	Regular	Flow Reversible	Flow Adaptive	Flow Regular
2008-07-04 06:00:00	141	141	0.0	50.0	4-2	3-3	3-3	0.12	0.18	0.18
2008-07-04 07:00:00	373	541	18.38	59.19	4-2	4-2	3-3	0.16	0.16	0.15
2008-07-04 08:00:00	405	763	30.65	65.33	4-2	4-2	3-3	0.18	0.18	0.12
2008-07-07 05:00:00	62	40	21.57	39.22	4-2	2-4	3-3	0.07	0.17	0.14
2008-07-07 06:00:00	127	142	5.58	52.79	4-2	3-3	3-3	0.13	0.18	0.18
2008-07-07 07:00:00	305	419	15.75	57.87	4-2	4-2	3-3	0.15	0.15	0.16
2008-07-07 08:00:00	327	603	29.68	64.84	4-2	4-2	3-3	0.18	0.18	0.13
2008-07-08 05:00:00	46	38	9.52	45.24	4-2	3-3	3-3	0.09	0.17	0.17
2008-07-08 06:00:00	124	122	0.81	49.59	4-2	3-3	3-3	0.11	0.18	0.18
2008-07-08 07:00:00	300	396	13.79	56.9	4-2	3-3	3-3	0.15	0.16	0.16
2008-07-08 08:00:00	347	583	25.38	62.69	4-2	4-2	3-3	0.17	0.17	0.14
2008-07-09 05:00:00	45	33	15.38	42.31	4-2	3-3	3-3	0.08	0.16	0.16
2008-07-09 06:00:00	118	111	3.06	48.47	4-2	3-3	3-3	0.11	0.18	0.18
2008-07-09 07:00:00	278	379	15.37	57.69	4-2	4-2	3-3	0.15	0.15	0.16
2008-07-09 08:00:00	375	584	21.79	60.9	4-2	4-2	3-3	0.17	0.17	0.15
2008-07-10 05:00:00	38	39	1.3	50.65	4-2	3-3	3-3	0.12	0.18	0.18
2008-07-10 06:00:00	122	125	1.21	50.61	4-2	3-3	3-3	0.12	0.18	0.18
2008-07-10 07:00:00	298	394	13.87	56.94	4-2	3-3	3-3	0.15	0.16	0.16
2008-07-10 08:00:00	354	616	27.01	63.51	4-2	4-2	3-3	0.18	0.18	0.13
2008-07-11 05:00:00	46	34	15.0	42.5	4-2	3-3	3-3	0.08	0.16	0.16
2008-07-11 06:00:00	94	108	6.93	53.47	4-2	3-3	3-3	0.13	0.17	0.17
2008-07-11 07:00:00	283	343	9.58	54.79	4-2	3-3	3-3	0.14	0.17	0.17
2008-07-11 08:00:00	321	576	28.43	64.21	4-2	4-2	3-3	0.18	0.18	0.13
2008-07-14 05:00:00	54	29	30.12	34.94	4-2	2-4	3-3	0.04	0.18	0.12
2008-07-14 06:00:00	123	143	7.52	53.76	4-2	3-3	3-3	0.13	0.17	0.17
2008-07-14 07:00:00	301	474	22.32	61.16	4-2	4-2	3-3	0.17	0.17	0.14
2008-07-14 08:00:00	339	733	36.75	68.38	4-2	4-2	3-3	0.17	0.17	0.1
2008-07-15 05:00:00	45	34	13.92	43.04	4-2	3-3	3-3	0.08	0.16	0.16
2008-07-15 06:00:00	131	159	9.66	54.83	4-2	3-3	3-3	0.14	0.17	0.17
2008-07-15 07:00:00	340	512	20.19	60.09	4-2	4-2	3-3	0.17	0.17	0.15
2008-07-15 08:00:00	404	735	29.06	64.53	4-2	4-2	3-3	0.18	0.18	0.13
2008-07-16 05:00:00	52	47	5.05	47.47	4-2	3-3	3-3	0.1	0.17	0.17
2008-07-16 06:00:00	150	154	1.32	50.66	4-2	3-3	3-3	0.12	0.18	0.18
2008-07-16 07:00:00	357	531	19.59	59.8	4-2	4-2	3-3	0.16	0.16	0.15
2008-07-16 08:00:00	415	757	29.18	64.59	4-2	4-2	3-3	0.18	0.18	0.13
2008-07-17 05:00:00	48	43	5.49	47.25	4-2	3-3	3-3	0.1	0.17	0.17
2008-07-17 06:00:00	158	150	2.6	48.7	4-2	3-3	3-3	0.11	0.18	0.18
2008-07-17 07:00:00	334	534	23.04	61.52	4-2	4-2	3-3	0.17	0.17	0.14
2008-07-17 08:00:00	372	693	30.14	65.07	4-2	4-2	3-3	0.18	0.18	0.12
2008-07-18 05:00:00	39	40	1.27	50.63	4-2	3-3	3-3	0.12	0.18	0.18
2008-07-18 06:00:00	113	130	7.0	53.5	4-2	3-3	3-3	0.13	0.17	0.17

Table 1: Example of a single processed file. Data from 2008 of a sensor located in Dunmore (N77-2).

Date	Vol North	Vol South	Asymmetry	South	Reversible	Adaptive	Regular	Flow Reversible	Flow Adaptive	Flow Regular
2008-07-18 07:00:00	284	371	13.28	56.64	4-2	3-3	3-3	0.15	0.16	0.16
2008-07-18 08:00:00	339	589	26.94	63.47	4-2	4-2	3-3	0.18	0.18	0.13
2008-07-21 05:00:00	54	42	12.5	43.75	4-2	3-3	3-3	0.08	0.16	0.16
2008-07-21 06:00:00	111	132	8.64	54.32	4-2	3-3	3-3	0.14	0.17	0.17
2008-07-21 07:00:00	312	327	2.35	51.17	4-2	3-3	3-3	0.12	0.18	0.18
2008-07-21 08:00:00	342	570	25.0	62.5	4-2	4-2	3-3	0.17	0.17	0.14
2008-07-22 05:00:00	38	45	8.43	54.22	4-2	3-3	3-3	0.14	0.17	0.17
2008-07-22 06:00:00	119	102	7.69	46.15	4-2	3-3	3-3	0.1	0.17	0.17
2008-07-22 07:00:00	273	339	10.78	55.39	4-2	3-3	3-3	0.14	0.17	0.17
2008-07-22 08:00:00	369	531	18.0	59.0	4-2	4-2	3-3	0.16	0.16	0.15
2008-07-23 05:00:00	39	34	6.85	46.58	4-2	3-3	3-3	0.1	0.17	0.17
2008-07-23 06:00:00	117	104	5.88	47.06	4-2	3-3	3-3	0.1	0.17	0.17
2008-07-23 07:00:00	292	322	4.89	52.44	4-2	3-3	3-3	0.13	0.18	0.18
2008-07-23 08:00:00	367	570	21.66	60.83	4-2	4-2	3-3	0.17	0.17	0.15
2008-07-24 05:00:00	33	38	7.04	53.52	4-2	3-3	3-3	0.13	0.17	0.17
2008-07-24 06:00:00	97	99	1.02	50.51	4-2	3-3	3-3	0.12	0.18	0.18
2008-07-24 07:00:00	289	356	10.39	55.19	4-2	3-3	3-3	0.14	0.17	0.17
2008-07-24 08:00:00	365	541	19.43	59.71	4-2	4-2	3-3	0.16	0.16	0.15
2008-07-25 05:00:00	35	37	2.78	51.39	4-2	3-3	3-3	0.12	0.18	0.18
2008-07-25 06:00:00	106	94	6.0	47.0	4-2	3-3	3-3	0.1	0.17	0.17
2008-07-25 07:00:00	258	319	10.57	55.29	4-2	3-3	3-3	0.14	0.17	0.17
2008-07-25 08:00:00	318	532	25.18	62.59	4-2	4-2	3-3	0.17	0.17	0.14
2008-07-28 05:00:00	49	31	22.5	38.75	4-2	2-4	3-3	0.06	0.17	0.14
2008-07-28 06:00:00	108	100	3.85	48.08	4-2	3-3	3-3	0.11	0.18	0.18
2008-07-28 07:00:00	278	311	5.6	52.8	4-2	3-3	3-3	0.13	0.18	0.18
2008-07-28 08:00:00	314	508	23.6	61.8	4-2	4-2	3-3	0.17	0.17	0.14
2008-07-29 05:00:00	45	29	21.62	39.19	4-2	2-4	3-3	0.07	0.17	0.14
2008-07-29 06:00:00	96	84	6.67	46.67	4-2	3-3	3-3	0.1	0.17	0.17
2008-07-29 07:00:00	265	312	8.15	54.07	4-2	3-3	3-3	0.14	0.17	0.17
2008-07-29 08:00:00	340	510	20.0	60.0	4-2	4-2	3-3	0.17	0.17	0.15
2008-07-30 05:00:00	41	46	5.75	52.87	4-2	3-3	3-3	0.13	0.18	0.18
2008-07-30 06:00:00	118	87	15.12	42.44	4-2	3-3	3-3	0.08	0.16	0.16
2008-07-30 07:00:00	292	340	7.59	53.8	4-2	3-3	3-3	0.13	0.17	0.17
2008-07-30 08:00:00	364	548	20.18	60.09	4-2	4-2	3-3	0.17	0.17	0.15
2008-07-31 05:00:00	47	31	20.51	39.74	4-2	2-4	3-3	0.07	0.17	0.14
2008-07-31 06:00:00	100	101	0.5	50.25	4-2	3-3	3-3	0.12	0.18	0.18
2008-07-31 07:00:00	273	328	9.15	54.58	4-2	3-3	3-3	0.14	0.17	0.17
2008-07-31 08:00:00	347	532	21.05	60.52	4-2	4-2	3-3	0.17	0.17	0.15
2008-08-01 05:00:00	47	41	6.82	46.59	4-2	3-3	3-3	0.1	0.17	0.17
2008-08-01 06:00:00	105	75	16.67	41.67	4-2	3-3	3-3	0.07	0.15	0.15
2008-08-01 07:00:00	213	305	17.76	58.88	4-2	4-2	3-3	0.16	0.16	0.16

Table 1: Example of a single processed file. Data from 2008 of a sensor located in Dunmore (N77-2).

Date	Vol North	Vol South	Asymmetry	South	Reversible	Adaptive	Regular	Flow Reversible	Flow Adaptive	Flow Regular
2008-08-01 08:00:00	297	504	25.84	62.92	4-2	4-2	3-3	0.17	0.17	0.14
2008-08-04 05:00:00	16	14	6.67	46.67	4-2	3-3	3-3	0.1	0.17	0.17
2008-08-04 06:00:00	23	21	4.55	47.73	4-2	3-3	3-3	0.1	0.17	0.17
2008-08-04 07:00:00	69	56	10.4	44.8	4-2	3-3	3-3	0.09	0.16	0.16
2008-08-04 08:00:00	85	82	1.8	49.1	4-2	3-3	3-3	0.11	0.18	0.18
2008-08-05 05:00:00	54	36	20.0	40.0	4-2	2-4	3-3	0.07	0.17	0.15
2008-08-05 06:00:00	112	109	1.36	49.32	4-2	3-3	3-3	0.11	0.18	0.18
2008-08-05 07:00:00	291	375	12.61	56.31	4-2	3-3	3-3	0.15	0.16	0.16
2008-08-05 08:00:00	364	490	14.75	57.38	4-2	4-2	3-3	0.15	0.15	0.16
2008-08-06 05:00:00	43	28	21.13	39.44	4-2	2-4	3-3	0.07	0.17	0.14
2008-08-06 06:00:00	115	110	2.22	48.89	4-2	3-3	3-3	0.11	0.18	0.18
2008-08-06 07:00:00	258	361	16.64	58.32	4-2	4-2	3-3	0.16	0.16	0.16
2008-08-06 08:00:00	330	520	22.35	61.18	4-2	4-2	3-3	0.17	0.17	0.14
2008-08-07 05:00:00	39	33	8.33	45.83	4-2	3-3	3-3	0.09	0.17	0.17
2008-08-07 06:00:00	119	114	2.15	48.93	4-2	3-3	3-3	0.11	0.18	0.18
2008-08-07 07:00:00	259	395	20.8	60.4	4-2	4-2	3-3	0.17	0.17	0.15
2008-08-07 08:00:00	361	537	19.6	59.8	4-2	4-2	3-3	0.16	0.16	0.15
2008-08-08 05:00:00	46	34	15.0	42.5	4-2	3-3	3-3	0.08	0.16	0.16
2008-08-08 06:00:00	99	121	10.0	55.0	4-2	3-3	3-3	0.14	0.17	0.17
2008-08-08 07:00:00	269	351	13.23	56.61	4-2	3-3	3-3	0.15	0.16	0.16
2008-08-08 08:00:00	320	523	24.08	62.04	4-2	4-2	3-3	0.17	0.17	0.14
2008-08-11 05:00:00	53	32	24.71	37.65	4-2	2-4	3-3	0.05	0.18	0.13
2008-08-11 06:00:00	138	97	17.45	41.28	4-2	3-3	3-3	0.07	0.15	0.15
2008-08-11 07:00:00	291	394	15.04	57.52	4-2	4-2	3-3	0.15	0.15	0.16
2008-08-11 08:00:00	288	522	28.89	64.44	4-2	4-2	3-3	0.18	0.18	0.13
2008-08-12 05:00:00	42	28	20.0	40.0	4-2	2-4	3-3	0.07	0.17	0.15
2008-08-12 06:00:00	121	113	3.42	48.29	4-2	3-3	3-3	0.11	0.18	0.18
2008-08-12 07:00:00	275	371	14.86	57.43	4-2	4-2	3-3	0.15	0.15	0.16
2008-08-12 08:00:00	338	559	24.64	62.32	4-2	4-2	3-3	0.17	0.17	0.14
2008-08-13 05:00:00	51	34	20.0	40.0	4-2	2-4	3-3	0.07	0.17	0.15
2008-08-13 06:00:00	123	110	5.58	47.21	4-2	3-3	3-3	0.1	0.17	0.17
2008-08-13 07:00:00	282	421	19.77	59.89	4-2	4-2	3-3	0.16	0.16	0.15
2008-08-13 08:00:00	321	543	25.69	62.85	4-2	4-2	3-3	0.17	0.17	0.14
2008-08-14 05:00:00	46	29	22.67	38.67	4-2	2-4	3-3	0.06	0.17	0.14
2008-08-14 06:00:00	103	114	5.07	52.53	4-2	3-3	3-3	0.13	0.18	0.18
2008-08-14 07:00:00	271	420	21.56	60.78	4-2	4-2	3-3	0.17	0.17	0.15
2008-08-14 08:00:00	360	567	22.33	61.17	4-2	4-2	3-3	0.17	0.17	0.14
2008-08-15 05:00:00	47	29	23.68	38.16	4-2	2-4	3-3	0.06	0.17	0.14
2008-08-15 06:00:00	107	85	11.46	44.27	4-2	3-3	3-3	0.09	0.16	0.16
2008-08-15 07:00:00	271	396	18.74	59.37	4-2	4-2	3-3	0.16	0.16	0.15
2008-08-15 08:00:00	309	536	26.86	63.43	4-2	4-2	3-3	0.18	0.18	0.13

Table 1: Example of a single processed file. Data from 2008 of a sensor located in Dunmore (N77-2).

Date	Vol North	Vol South	Asymmetry	South	Reversible	Adaptive	Regular	Flow Reversible	Flow Adaptive	Flow Regular
2008-08-18 05:00:00	60	32	30.43	34.78	4-2	2-4	3-3	0.04	0.18	0.12
2008-08-18 06:00:00	123	112	4.68	47.66	4-2	3-3	3-3	0.1	0.17	0.17
2008-08-18 07:00:00	293	392	14.45	57.23	4-2	4-2	3-3	0.15	0.15	0.16
2008-08-18 08:00:00	314	548	27.15	63.57	4-2	4-2	3-3	0.18	0.18	0.13
2008-08-19 05:00:00	40	35	6.67	46.67	4-2	3-3	3-3	0.1	0.17	0.17
2008-08-19 06:00:00	111	112	0.45	50.22	4-2	3-3	3-3	0.12	0.18	0.18
2008-08-19 07:00:00	296	437	19.24	59.62	4-2	4-2	3-3	0.16	0.16	0.15
2008-08-19 08:00:00	373	586	22.21	61.11	4-2	4-2	3-3	0.17	0.17	0.14
2008-08-20 05:00:00	40	46	6.98	53.49	4-2	3-3	3-3	0.13	0.17	0.17
2008-08-20 06:00:00	112	109	1.36	49.32	4-2	3-3	3-3	0.11	0.18	0.18
2008-08-20 07:00:00	324	420	12.9	56.45	4-2	3-3	3-3	0.15	0.16	0.16
2008-08-20 08:00:00	378	613	23.71	61.86	4-2	4-2	3-3	0.17	0.17	0.14
2008-08-21 05:00:00	54	35	21.35	39.33	4-2	2-4	3-3	0.07	0.17	0.14
2008-08-21 06:00:00	106	116	4.5	52.25	4-2	3-3	3-3	0.13	0.18	0.18
2008-08-21 07:00:00	315	399	11.76	55.88	4-2	3-3	3-3	0.14	0.17	0.17
2008-08-21 08:00:00	359	562	22.04	61.02	4-2	4-2	3-3	0.17	0.17	0.14
2008-08-22 05:00:00	43	45	2.27	51.14	4-2	3-3	3-3	0.12	0.18	0.18
2008-08-22 06:00:00	114	107	3.17	48.42	4-2	3-3	3-3	0.11	0.18	0.18
2008-08-22 07:00:00	304	379	10.98	55.49	4-2	3-3	3-3	0.14	0.17	0.17
2008-08-22 08:00:00	332	558	25.39	62.7	4-2	4-2	3-3	0.17	0.17	0.14
2008-08-25 05:00:00	58	30	31.82	34.09	4-2	2-4	3-3	0.04	0.18	0.12
2008-08-25 06:00:00	138	133	1.85	49.08	4-2	3-3	3-3	0.11	0.18	0.18
2008-08-25 07:00:00	351	411	7.87	53.94	4-2	3-3	3-3	0.13	0.17	0.17
2008-08-25 08:00:00	374	573	21.01	60.51	4-2	4-2	3-3	0.17	0.17	0.15
2008-08-26 05:00:00	34	30	6.25	46.88	4-2	3-3	3-3	0.1	0.17	0.17
2008-08-26 06:00:00	130	117	5.26	47.37	4-2	3-3	3-3	0.1	0.17	0.17
2008-08-26 07:00:00	360	384	3.23	51.61	4-2	3-3	3-3	0.12	0.18	0.18
2008-08-26 08:00:00	393	578	19.05	59.53	4-2	4-2	3-3	0.16	0.16	0.15
2008-08-27 05:00:00	42	40	2.44	48.78	4-2	3-3	3-3	0.11	0.18	0.18
2008-08-27 06:00:00	127	129	0.78	50.39	4-2	3-3	3-3	0.12	0.18	0.18
2008-08-27 07:00:00	339	396	7.76	53.88	4-2	3-3	3-3	0.13	0.17	0.17
2008-08-27 08:00:00	377	661	27.36	63.68	4-2	4-2	3-3	0.18	0.18	0.13
2008-08-28 05:00:00	36	42	7.69	53.85	4-2	3-3	3-3	0.13	0.17	0.17
2008-08-28 06:00:00	130	149	6.81	53.41	4-2	3-3	3-3	0.13	0.17	0.17
2008-08-28 07:00:00	333	410	10.36	55.18	4-2	3-3	3-3	0.14	0.17	0.17
2008-08-28 08:00:00	404	623	21.32	60.66	4-2	4-2	3-3	0.17	0.17	0.15
2008-08-29 05:00:00	36	36	0.0	50.0	4-2	3-3	3-3	0.12	0.18	0.18
2008-08-29 06:00:00	116	114	0.87	49.57	4-2	3-3	3-3	0.11	0.18	0.18
2008-08-29 07:00:00	312	419	14.64	57.32	4-2	4-2	3-3	0.15	0.15	0.16
2008-08-29 08:00:00	432	579	14.54	57.27	4-2	4-2	3-3	0.15	0.15	0.16
2008-09-01 05:00:00	48	44	4.35	47.83	4-2	3-3	3-3	0.1	0.17	0.17

Table 1: Example of a single processed file. Data from 2008 of a sensor located in Dunmore (N77-2).

Date	Vol North	Vol South	Asymmetry	South	Reversible	Adaptive	Regular	Flow Reversible	Flow Adaptive	Flow Regular
2008-09-01 06:00:00	129	125	1.57	49.21	4-2	3-3	3-3	0.11	0.18	0.18
2008-09-01 07:00:00	337	450	14.36	57.18	4-2	4-2	3-3	0.15	0.15	0.16
2008-09-01 08:00:00	403	645	23.09	61.55	4-2	4-2	3-3	0.17	0.17	0.14
2008-09-02 05:00:00	38	31	10.14	44.93	4-2	3-3	3-3	0.09	0.16	0.16
2008-09-02 06:00:00	113	129	6.61	53.31	4-2	3-3	3-3	0.13	0.17	0.17
2008-09-02 07:00:00	341	442	12.9	56.45	4-2	3-3	3-3	0.15	0.16	0.16
2008-09-02 08:00:00	391	644	24.44	62.22	4-2	4-2	3-3	0.17	0.17	0.14
2008-09-03 05:00:00	43	39	4.88	47.56	4-2	3-3	3-3	0.1	0.17	0.17
2008-09-03 06:00:00	110	127	7.17	53.59	4-2	3-3	3-3	0.13	0.17	0.17
2008-09-03 07:00:00	335	428	12.19	56.09	4-2	3-3	3-3	0.15	0.16	0.16
2008-09-03 08:00:00	422	642	20.68	60.34	4-2	4-2	3-3	0.17	0.17	0.15
2008-09-04 05:00:00	40	40	0.0	50.0	4-2	3-3	3-3	0.12	0.18	0.18
2008-09-04 06:00:00	109	145	14.17	57.09	4-2	4-2	3-3	0.15	0.15	0.16
2008-09-04 07:00:00	307	467	20.67	60.34	4-2	4-2	3-3	0.17	0.17	0.15
2008-09-04 08:00:00	406	656	23.54	61.77	4-2	4-2	3-3	0.17	0.17	0.14
2008-09-05 05:00:00	52	36	18.18	40.91	4-2	2-4	3-3	0.07	0.17	0.15
2008-09-05 06:00:00	91	122	14.55	57.28	4-2	4-2	3-3	0.15	0.15	0.16
2008-09-05 07:00:00	300	398	14.04	57.02	4-2	4-2	3-3	0.15	0.15	0.16
2008-09-05 08:00:00	383	575	20.04	60.02	4-2	4-2	3-3	0.17	0.17	0.15
2008-09-08 05:00:00	32	32	0.0	50.0	4-2	3-3	3-3	0.12	0.18	0.18
2008-09-08 06:00:00	99	108	4.35	52.17	4-2	3-3	3-3	0.13	0.18	0.18
2008-09-08 07:00:00	281	392	16.49	58.25	4-2	4-2	3-3	0.16	0.16	0.16
2008-09-08 08:00:00	347	566	23.99	61.99	4-2	4-2	3-3	0.17	0.17	0.14
2008-09-09 05:00:00	43	36	8.86	45.57	4-2	3-3	3-3	0.09	0.17	0.17
2008-09-09 06:00:00	108	101	3.35	48.33	4-2	3-3	3-3	0.11	0.18	0.18
2008-09-09 07:00:00	302	388	12.46	56.23	4-2	3-3	3-3	0.15	0.16	0.16
2008-09-09 08:00:00	417	639	21.02	60.51	4-2	4-2	3-3	0.17	0.17	0.15
2008-09-10 05:00:00	50	40	11.11	44.44	4-2	3-3	3-3	0.09	0.16	0.16
2008-09-10 06:00:00	119	102	7.69	46.15	4-2	3-3	3-3	0.1	0.17	0.17
2008-09-10 07:00:00	332	469	17.1	58.55	4-2	4-2	3-3	0.16	0.16	0.16
2008-09-10 08:00:00	418	628	20.08	60.04	4-2	4-2	3-3	0.17	0.17	0.15
2008-09-11 05:00:00	46	37	10.84	44.58	4-2	3-3	3-3	0.09	0.16	0.16
2008-09-11 06:00:00	111	122	4.72	52.36	4-2	3-3	3-3	0.13	0.18	0.18
2008-09-11 07:00:00	332	442	14.21	57.11	4-2	4-2	3-3	0.15	0.15	0.16
2008-09-11 08:00:00	439	692	22.37	61.18	4-2	4-2	3-3	0.17	0.17	0.14
2008-09-12 05:00:00	46	42	4.55	47.73	4-2	3-3	3-3	0.1	0.17	0.17
2008-09-12 06:00:00	114	116	0.87	50.43	4-2	3-3	3-3	0.12	0.18	0.18
2008-09-12 07:00:00	286	405	17.22	58.61	4-2	4-2	3-3	0.16	0.16	0.16
2008-09-12 08:00:00	391	615	22.27	61.13	4-2	4-2	3-3	0.17	0.17	0.14
2008-09-15 05:00:00	57	22	44.3	27.85	4-2	2-4	3-3	0.02	0.15	0.08
2008-09-15 06:00:00	129	119	4.03	47.98	4-2	3-3	3-3	0.1	0.17	0.17

Table 1: Example of a single processed file. Data from 2008 of a sensor located in Dunmore (N77-2).

Date	Vol North	Vol South	Asymmetry	South	Reversible	Adaptive	Regular	Flow Reversible	Flow Adaptive	Flow Regular
2008-09-15 07:00:00	307	473	21.28	60.64	4-2	4-2	3-3	0.17	0.17	0.15
2008-09-15 08:00:00	416	676	23.81	61.9	4-2	4-2	3-3	0.17	0.17	0.14
2008-09-16 05:00:00	46	38	9.52	45.24	4-2	3-3	3-3	0.09	0.17	0.17
2008-09-16 06:00:00	123	124	0.4	50.2	4-2	3-3	3-3	0.12	0.18	0.18
2008-09-16 07:00:00	335	433	12.76	56.38	4-2	3-3	3-3	0.15	0.16	0.16
2008-09-16 08:00:00	414	652	22.33	61.16	4-2	4-2	3-3	0.17	0.17	0.14
2008-09-17 05:00:00	45	39	7.14	46.43	4-2	3-3	3-3	0.1	0.17	0.17
2008-09-17 06:00:00	108	133	10.37	55.19	4-2	3-3	3-3	0.14	0.17	0.17
2008-09-17 07:00:00	320	424	13.98	56.99	4-2	3-3	3-3	0.15	0.16	0.16
2008-09-17 08:00:00	449	667	19.53	59.77	4-2	4-2	3-3	0.16	0.16	0.15
2008-09-18 05:00:00	42	38	5.0	47.5	4-2	3-3	3-3	0.1	0.17	0.17
2008-09-18 06:00:00	102	142	16.39	58.2	4-2	4-2	3-3	0.16	0.16	0.16
2008-09-18 07:00:00	347	425	10.1	55.05	4-2	3-3	3-3	0.14	0.17	0.17
2008-09-18 08:00:00	406	642	22.52	61.26	4-2	4-2	3-3	0.17	0.17	0.14
2008-09-19 05:00:00	49	41	8.89	45.56	4-2	3-3	3-3	0.09	0.17	0.17
2008-09-19 06:00:00	101	122	9.42	54.71	4-2	3-3	3-3	0.14	0.17	0.17
2008-09-19 07:00:00	305	391	12.36	56.18	4-2	3-3	3-3	0.15	0.16	0.16
2008-09-19 08:00:00	395	642	23.82	61.91	4-2	4-2	3-3	0.17	0.17	0.14
2008-09-22 05:00:00	65	48	15.04	42.48	4-2	3-3	3-3	0.08	0.16	0.16
2008-09-22 06:00:00	130	116	5.69	47.15	4-2	3-3	3-3	0.1	0.17	0.17
2008-09-22 07:00:00	311	459	19.22	59.61	4-2	4-2	3-3	0.16	0.16	0.15
2008-09-22 08:00:00	396	703	27.93	63.97	4-2	4-2	3-3	0.18	0.18	0.13
2008-09-23 05:00:00	42	78	30.0	65.0	4-2	4-2	3-3	0.18	0.18	0.12
2008-09-23 06:00:00	131	325	42.54	71.27	4-2	4-2	3-3	0.16	0.16	0.09
2008-09-23 07:00:00	290	1018	55.66	77.83	4-2	4-2	3-3	0.12	0.12	0.06
2008-09-23 08:00:00	389	1002	44.07	72.03	4-2	4-2	3-3	0.16	0.16	0.08
2008-09-24 05:00:00	47	81	26.56	63.28	4-2	4-2	3-3	0.18	0.18	0.13
2008-09-24 06:00:00	110	523	65.24	82.62	4-2	4-2	3-3	0.09	0.09	0.03
2008-09-24 07:00:00	315	1122	56.16	78.08	4-2	4-2	3-3	0.12	0.12	0.05
2008-09-24 08:00:00	437	908	35.02	67.51	4-2	4-2	3-3	0.18	0.18	0.11
2008-09-25 05:00:00	51	78	20.93	60.47	4-2	4-2	3-3	0.17	0.17	0.15
2008-09-25 06:00:00	123	423	54.95	77.47	4-2	4-2	3-3	0.12	0.12	0.06
2008-09-25 07:00:00	300	982	53.2	76.6	4-2	4-2	3-3	0.13	0.13	0.06
2008-09-25 08:00:00	378	889	40.33	70.17	4-2	4-2	3-3	0.17	0.17	0.09
2008-09-26 05:00:00	56	44	12.0	44.0	4-2	3-3	3-3	0.09	0.16	0.16
2008-09-26 06:00:00	120	130	4.0	52.0	4-2	3-3	3-3	0.13	0.18	0.18
2008-09-26 07:00:00	292	378	12.84	56.42	4-2	3-3	3-3	0.15	0.16	0.16
2008-09-26 08:00:00	409	626	20.97	60.48	4-2	4-2	3-3	0.17	0.17	0.15
2008-09-29 05:00:00	59	46	12.38	43.81	4-2	3-3	3-3	0.08	0.16	0.16
2008-09-29 06:00:00	132	122	3.94	48.03	4-2	3-3	3-3	0.11	0.18	0.18
2008-09-29 07:00:00	320	467	18.68	59.34	4-2	4-2	3-3	0.16	0.16	0.15

Table 1: Example of a single processed file. Data from 2008 of a sensor located in Dunmore (N77-2).

Date	Vol North	Vol South	Asymmetry	South	Reversible	Adaptive	Regular	Flow Reversible	Flow Adaptive	Flow Regular
2008-09-29 08:00:00	405	686	25.76	62.88	4-2	4-2	3-3	0.17	0.17	0.14
2008-09-30 05:00:00	42	33	12.0	44.0	4-2	3-3	3-3	0.09	0.16	0.16
2008-09-30 06:00:00	120	135	5.88	52.94	4-2	3-3	3-3	0.13	0.18	0.18
2008-09-30 07:00:00	311	418	14.68	57.34	4-2	4-2	3-3	0.15	0.15	0.16
2008-09-30 08:00:00	392	656	25.19	62.6	4-2	4-2	3-3	0.17	0.17	0.14
2008-10-01 05:00:00	47	44	3.3	48.35	4-2	3-3	3-3	0.11	0.18	0.18
2008-10-01 06:00:00	130	109	8.79	45.61	4-2	3-3	3-3	0.09	0.17	0.17
2008-10-01 07:00:00	323	393	9.78	54.89	4-2	3-3	3-3	0.14	0.17	0.17
2008-10-01 08:00:00	384	653	25.94	62.97	4-2	4-2	3-3	0.17	0.17	0.14
2008-10-02 05:00:00	55	42	13.4	43.3	4-2	3-3	3-3	0.08	0.16	0.16
2008-10-02 06:00:00	128	129	0.39	50.19	4-2	3-3	3-3	0.12	0.18	0.18
2008-10-02 07:00:00	328	426	13.0	56.5	4-2	3-3	3-3	0.15	0.16	0.16
2008-10-02 08:00:00	430	612	17.47	58.73	4-2	4-2	3-3	0.16	0.16	0.16
2008-10-03 05:00:00	43	33	13.16	43.42	4-2	3-3	3-3	0.08	0.16	0.16
2008-10-03 06:00:00	121	121	0.0	50.0	4-2	3-3	3-3	0.12	0.18	0.18
2008-10-03 07:00:00	319	394	10.52	55.26	4-2	3-3	3-3	0.14	0.17	0.17
2008-10-03 08:00:00	378	625	24.63	62.31	4-2	4-2	3-3	0.17	0.17	0.14
2008-10-06 05:00:00	57	35	23.91	38.04	4-2	2-4	3-3	0.06	0.17	0.14
2008-10-06 06:00:00	146	110	14.06	42.97	4-2	3-3	3-3	0.08	0.16	0.16
2008-10-06 07:00:00	318	432	15.2	57.6	4-2	4-2	3-3	0.15	0.15	0.16
2008-10-06 08:00:00	423	628	19.51	59.75	4-2	4-2	3-3	0.16	0.16	0.15
2008-10-07 05:00:00	46	24	31.43	34.29	4-2	2-4	3-3	0.04	0.18	0.12
2008-10-07 06:00:00	119	132	5.18	52.59	4-2	3-3	3-3	0.13	0.18	0.18
2008-10-07 07:00:00	321	442	15.86	57.93	4-2	4-2	3-3	0.15	0.15	0.16
2008-10-07 08:00:00	440	631	17.83	58.92	4-2	4-2	3-3	0.16	0.16	0.16
2008-10-08 05:00:00	35	36	1.41	50.7	4-2	3-3	3-3	0.12	0.18	0.18
2008-10-08 06:00:00	124	118	2.48	48.76	4-2	3-3	3-3	0.11	0.18	0.18
2008-10-08 07:00:00	329	413	11.32	55.66	4-2	3-3	3-3	0.14	0.17	0.17
2008-10-08 08:00:00	391	603	21.33	60.66	4-2	4-2	3-3	0.17	0.17	0.15
2008-10-09 05:00:00	44	33	14.29	42.86	4-2	3-3	3-3	0.08	0.16	0.16
2008-10-09 06:00:00	120	125	2.04	51.02	4-2	3-3	3-3	0.12	0.18	0.18
2008-10-09 07:00:00	321	432	14.74	57.37	4-2	4-2	3-3	0.15	0.15	0.16
2008-10-09 08:00:00	424	634	19.85	59.92	4-2	4-2	3-3	0.16	0.16	0.15
2008-10-10 05:00:00	44	34	12.82	43.59	4-2	3-3	3-3	0.08	0.16	0.16
2008-10-10 06:00:00	102	102	0.0	50.0	4-2	3-3	3-3	0.12	0.18	0.18
2008-10-10 07:00:00	313	399	12.08	56.04	4-2	3-3	3-3	0.15	0.16	0.16
2008-10-10 08:00:00	391	604	21.41	60.7	4-2	4-2	3-3	0.17	0.17	0.15
2008-10-13 05:00:00	52	36	18.18	40.91	4-2	2-4	3-3	0.07	0.17	0.15
2008-10-13 06:00:00	144	116	10.77	44.62	4-2	3-3	3-3	0.09	0.16	0.16
2008-10-13 07:00:00	334	425	11.99	55.99	4-2	3-3	3-3	0.14	0.17	0.17
2008-10-13 08:00:00	408	588	18.07	59.04	4-2	4-2	3-3	0.16	0.16	0.15

Table 1: Example of a single processed file. Data from 2008 of a sensor located in Dunmore (N77-2).

Date	Vol North	Vol South	Asymmetry	South	Reversible	Adaptive	Regular	Flow Reversible	Flow Adaptive	Flow Regular
2008-10-14 05:00:00	42	28	20.0	40.0	4-2	2-4	3-3	0.07	0.17	0.15
2008-10-14 06:00:00	111	112	0.45	50.22	4-2	3-3	3-3	0.12	0.18	0.18
2008-10-14 07:00:00	312	391	11.24	55.62	4-2	3-3	3-3	0.14	0.17	0.17
2008-10-14 08:00:00	392	571	18.59	59.29	4-2	4-2	3-3	0.16	0.16	0.15
2008-10-15 05:00:00	45	45	0.0	50.0	4-2	3-3	3-3	0.12	0.18	0.18
2008-10-15 06:00:00	105	114	4.11	52.05	4-2	3-3	3-3	0.13	0.18	0.18
2008-10-15 07:00:00	322	408	11.78	55.89	4-2	3-3	3-3	0.14	0.17	0.17
2008-10-15 08:00:00	385	577	19.96	59.98	4-2	4-2	3-3	0.16	0.16	0.15
2008-10-16 05:00:00	42	38	5.0	47.5	4-2	3-3	3-3	0.1	0.17	0.17
2008-10-16 06:00:00	123	123	0.0	50.0	4-2	3-3	3-3	0.12	0.18	0.18
2008-10-16 07:00:00	329	421	12.27	56.13	4-2	3-3	3-3	0.15	0.16	0.16
2008-10-16 08:00:00	369	607	24.39	62.19	4-2	4-2	3-3	0.17	0.17	0.14
2008-10-17 05:00:00	37	33	5.71	47.14	4-2	3-3	3-3	0.1	0.17	0.17
2008-10-17 06:00:00	102	92	5.15	47.42	4-2	3-3	3-3	0.1	0.17	0.17
2008-10-17 07:00:00	287	385	14.58	57.29	4-2	4-2	3-3	0.15	0.15	0.16
2008-10-17 08:00:00	360	537	19.73	59.87	4-2	4-2	3-3	0.16	0.16	0.15
2008-10-20 05:00:00	59	35	25.53	37.23	4-2	2-4	3-3	0.05	0.18	0.13
2008-10-20 06:00:00	136	116	7.94	46.03	4-2	3-3	3-3	0.1	0.17	0.17
2008-10-20 07:00:00	324	398	10.25	55.12	4-2	3-3	3-3	0.14	0.17	0.17
2008-10-20 08:00:00	416	666	23.11	61.55	4-2	4-2	3-3	0.17	0.17	0.14
2008-10-21 05:00:00	48	32	20.0	40.0	4-2	2-4	3-3	0.07	0.17	0.15
2008-10-21 06:00:00	118	107	4.89	47.56	4-2	3-3	3-3	0.1	0.17	0.17
2008-10-21 07:00:00	340	407	8.97	54.48	4-2	3-3	3-3	0.14	0.17	0.17
2008-10-21 08:00:00	445	638	17.82	58.91	4-2	4-2	3-3	0.16	0.16	0.16
2008-10-22 05:00:00	64	33	31.96	34.02	4-2	2-4	3-3	0.04	0.18	0.12
2008-10-22 06:00:00	134	113	8.5	45.75	4-2	3-3	3-3	0.09	0.17	0.17
2008-10-22 07:00:00	327	421	12.57	56.28	4-2	3-3	3-3	0.15	0.16	0.16
2008-10-22 08:00:00	385	662	26.46	63.23	4-2	4-2	3-3	0.18	0.18	0.13
2008-10-23 05:00:00	44	43	1.15	49.43	4-2	3-3	3-3	0.11	0.18	0.18
2008-10-23 06:00:00	127	110	7.17	46.41	4-2	3-3	3-3	0.1	0.17	0.17
2008-10-23 07:00:00	301	402	14.37	57.18	4-2	4-2	3-3	0.15	0.15	0.16
2008-10-23 08:00:00	376	622	24.65	62.32	4-2	4-2	3-3	0.17	0.17	0.14
2008-10-24 05:00:00	34	30	6.25	46.88	4-2	3-3	3-3	0.1	0.17	0.17
2008-10-24 06:00:00	122	86	17.31	41.35	4-2	3-3	3-3	0.07	0.15	0.15
2008-10-24 07:00:00	282	375	14.16	57.08	4-2	4-2	3-3	0.15	0.15	0.16
2008-10-24 08:00:00	402	591	19.03	59.52	4-2	4-2	3-3	0.16	0.16	0.15
2008-10-27 05:00:00	19	8	40.74	29.63	4-2	2-4	3-3	0.02	0.16	0.09
2008-10-27 06:00:00	22	15	18.92	40.54	4-2	2-4	3-3	0.07	0.17	0.15
2008-10-27 07:00:00	66	62	3.12	48.44	4-2	3-3	3-3	0.11	0.18	0.18
2008-10-27 08:00:00	100	71	16.96	41.52	4-2	3-3	3-3	0.07	0.15	0.15
2008-10-28 05:00:00	43	36	8.86	45.57	4-2	3-3	3-3	0.09	0.17	0.17

Table 1: Example of a single processed file. Data from 2008 of a sensor located in Dunmore (N77-2).

Date	Vol North	Vol South	Asymmetry	South	Reversible	Adaptive	Regular	Flow Reversible	Flow Adaptive	Flow Regular
2008-10-28 06:00:00	157	101	21.71	39.15	4-2	2-4	3-3	0.07	0.17	0.14
2008-10-28 07:00:00	298	422	17.22	58.61	4-2	4-2	3-3	0.16	0.16	0.16
2008-10-28 08:00:00	316	561	27.94	63.97	4-2	4-2	3-3	0.18	0.18	0.13
2008-10-29 05:00:00	37	33	5.71	47.14	4-2	3-3	3-3	0.1	0.17	0.17
2008-10-29 06:00:00	108	94	6.93	46.53	4-2	3-3	3-3	0.1	0.17	0.17
2008-10-29 07:00:00	290	369	11.99	55.99	4-2	3-3	3-3	0.14	0.17	0.17
2008-10-29 08:00:00	331	571	26.61	63.3	4-2	4-2	3-3	0.18	0.18	0.13
2008-10-30 05:00:00	43	44	1.15	50.57	4-2	3-3	3-3	0.12	0.18	0.18
2008-10-30 06:00:00	124	98	11.71	44.14	4-2	3-3	3-3	0.09	0.16	0.16
2008-10-30 07:00:00	301	394	13.38	56.69	4-2	3-3	3-3	0.15	0.16	0.16
2008-10-30 08:00:00	350	551	22.31	61.15	4-2	4-2	3-3	0.17	0.17	0.14
2008-10-31 05:00:00	24	29	9.43	54.72	4-2	3-3	3-3	0.14	0.17	0.17
2008-10-31 06:00:00	105	89	8.25	45.88	4-2	3-3	3-3	0.09	0.17	0.17
2008-10-31 07:00:00	256	361	17.02	58.51	4-2	4-2	3-3	0.16	0.16	0.16
2008-10-31 08:00:00	319	520	23.96	61.98	4-2	4-2	3-3	0.17	0.17	0.14
2008-11-03 05:00:00	56	36	21.74	39.13	4-2	2-4	3-3	0.07	0.17	0.14
2008-11-03 06:00:00	132	112	8.2	45.9	4-2	3-3	3-3	0.09	0.17	0.17
2008-11-03 07:00:00	331	421	11.97	55.98	4-2	3-3	3-3	0.14	0.17	0.17
2008-11-03 08:00:00	393	668	25.92	62.96	4-2	4-2	3-3	0.17	0.17	0.14
2008-11-04 05:00:00	53	31	26.19	36.9	4-2	2-4	3-3	0.05	0.18	0.13
2008-11-04 06:00:00	108	108	0.0	50.0	4-2	3-3	3-3	0.12	0.18	0.18
2008-11-04 07:00:00	343	389	6.28	53.14	4-2	3-3	3-3	0.13	0.17	0.17
2008-11-04 08:00:00	446	651	18.69	59.34	4-2	4-2	3-3	0.16	0.16	0.15
2008-11-05 05:00:00	45	33	15.38	42.31	4-2	3-3	3-3	0.08	0.16	0.16
2008-11-05 06:00:00	110	117	3.08	51.54	4-2	3-3	3-3	0.12	0.18	0.18
2008-11-05 07:00:00	340	395	7.48	53.74	4-2	3-3	3-3	0.13	0.17	0.17
2008-11-05 08:00:00	405	634	22.04	61.02	4-2	4-2	3-3	0.17	0.17	0.14
2008-11-06 05:00:00	50	38	13.64	43.18	4-2	3-3	3-3	0.08	0.16	0.16
2008-11-06 06:00:00	115	116	0.43	50.22	4-2	3-3	3-3	0.12	0.18	0.18
2008-11-06 07:00:00	361	416	7.08	53.54	4-2	3-3	3-3	0.13	0.17	0.17
2008-11-06 08:00:00	389	639	24.32	62.16	4-2	4-2	3-3	0.17	0.17	0.14
2008-11-07 05:00:00	43	35	10.26	44.87	4-2	3-3	3-3	0.09	0.16	0.16
2008-11-07 06:00:00	116	116	0.0	50.0	4-2	3-3	3-3	0.12	0.18	0.18
2008-11-07 07:00:00	314	388	10.54	55.27	4-2	3-3	3-3	0.14	0.17	0.17
2008-11-07 08:00:00	395	618	22.01	61.01	4-2	4-2	3-3	0.17	0.17	0.14
2008-11-10 05:00:00	67	34	32.67	33.66	4-2	2-4	3-3	0.04	0.18	0.11
2008-11-10 06:00:00	137	106	12.76	43.62	4-2	3-3	3-3	0.08	0.16	0.16
2008-11-10 07:00:00	323	437	15.0	57.5	4-2	4-2	3-3	0.15	0.15	0.16
2008-11-10 08:00:00	411	638	21.64	60.82	4-2	4-2	3-3	0.17	0.17	0.15
2008-11-11 05:00:00	47	36	13.25	43.37	4-2	3-3	3-3	0.08	0.16	0.16
2008-11-11 06:00:00	132	108	10.0	45.0	4-2	3-3	3-3	0.09	0.17	0.17

Table 1: Example of a single processed file. Data from 2008 of a sensor located in Dunmore (N77-2).

Date	Vol North	Vol South	Asymmetry	South	Reversible	Adaptive	Regular	Flow Reversible	Flow Adaptive	Flow Regular
2008-11-11 07:00:00	353	406	6.98	53.49	4-2	3-3	3-3	0.13	0.17	0.17
2008-11-11 08:00:00	402	659	24.22	62.11	4-2	4-2	3-3	0.17	0.17	0.14
2008-11-12 05:00:00	50	40	11.11	44.44	4-2	3-3	3-3	0.09	0.16	0.16
2008-11-12 06:00:00	116	106	4.5	47.75	4-2	3-3	3-3	0.1	0.17	0.17
2008-11-12 07:00:00	325	393	9.47	54.74	4-2	3-3	3-3	0.14	0.17	0.17
2008-11-12 08:00:00	414	646	21.89	60.94	4-2	4-2	3-3	0.17	0.17	0.15
2008-11-13 05:00:00	52	34	20.93	39.53	4-2	2-4	3-3	0.07	0.17	0.14
2008-11-13 06:00:00	119	110	3.93	48.03	4-2	3-3	3-3	0.11	0.18	0.18
2008-11-13 07:00:00	310	442	17.55	58.78	4-2	4-2	3-3	0.16	0.16	0.16
2008-11-13 08:00:00	417	665	22.92	61.46	4-2	4-2	3-3	0.17	0.17	0.14
2008-11-14 05:00:00	52	37	16.85	41.57	4-2	3-3	3-3	0.07	0.15	0.15
2008-11-14 06:00:00	125	100	11.11	44.44	4-2	3-3	3-3	0.09	0.16	0.16
2008-11-14 07:00:00	322	371	7.07	53.54	4-2	3-3	3-3	0.13	0.17	0.17
2008-11-14 08:00:00	375	627	25.15	62.57	4-2	4-2	3-3	0.17	0.17	0.14
2008-11-17 05:00:00	59	36	24.21	37.89	4-2	2-4	3-3	0.05	0.18	0.13
2008-11-17 06:00:00	148	119	10.86	44.57	4-2	3-3	3-3	0.09	0.16	0.16
2008-11-17 07:00:00	343	425	10.68	55.34	4-2	3-3	3-3	0.14	0.17	0.17
2008-11-17 08:00:00	395	651	24.47	62.24	4-2	4-2	3-3	0.17	0.17	0.14
2008-11-18 05:00:00	45	28	23.29	38.36	4-2	2-4	3-3	0.06	0.17	0.14
2008-11-18 06:00:00	140	120	7.69	46.15	4-2	3-3	3-3	0.1	0.17	0.17
2008-11-18 07:00:00	317	423	14.32	57.16	4-2	4-2	3-3	0.15	0.15	0.16
2008-11-18 08:00:00	457	670	18.9	59.45	4-2	4-2	3-3	0.16	0.16	0.15
2008-11-19 05:00:00	43	32	14.67	42.67	4-2	3-3	3-3	0.08	0.16	0.16
2008-11-19 06:00:00	148	108	15.62	42.19	4-2	3-3	3-3	0.08	0.16	0.16
2008-11-19 07:00:00	323	399	10.53	55.26	4-2	3-3	3-3	0.14	0.17	0.17
2008-11-19 08:00:00	432	658	20.73	60.37	4-2	4-2	3-3	0.17	0.17	0.15
2008-11-20 05:00:00	48	39	10.34	44.83	4-2	3-3	3-3	0.09	0.16	0.16
2008-11-20 06:00:00	118	111	3.06	48.47	4-2	3-3	3-3	0.11	0.18	0.18
2008-11-20 07:00:00	331	440	14.14	57.07	4-2	4-2	3-3	0.15	0.15	0.16
2008-11-20 08:00:00	405	654	23.51	61.76	4-2	4-2	3-3	0.17	0.17	0.14
2008-11-21 05:00:00	48	42	6.67	46.67	4-2	3-3	3-3	0.1	0.17	0.17
2008-11-21 06:00:00	118	101	7.76	46.12	4-2	3-3	3-3	0.1	0.17	0.17
2008-11-21 07:00:00	298	406	15.34	57.67	4-2	4-2	3-3	0.15	0.15	0.16
2008-11-21 08:00:00	420	628	19.85	59.92	4-2	4-2	3-3	0.16	0.16	0.15
2008-11-24 05:00:00	46	36	12.2	43.9	4-2	3-3	3-3	0.08	0.16	0.16
2008-11-24 06:00:00	132	108	10.0	45.0	4-2	3-3	3-3	0.09	0.17	0.17
2008-11-24 07:00:00	319	427	14.48	57.24	4-2	4-2	3-3	0.15	0.15	0.16
2008-11-24 08:00:00	413	685	24.77	62.39	4-2	4-2	3-3	0.17	0.17	0.14
2008-11-25 05:00:00	34	31	4.62	47.69	4-2	3-3	3-3	0.1	0.17	0.17
2008-11-25 06:00:00	110	109	0.46	49.77	4-2	3-3	3-3	0.11	0.18	0.18
2008-11-25 07:00:00	331	400	9.44	54.72	4-2	3-3	3-3	0.14	0.17	0.17

Table 1: Example of a single processed file. Data from 2008 of a sensor located in Dunmore (N77-2).

Date	Vol North	Vol South	Asymmetry	South	Reversible	Adaptive	Regular	Flow Reversible	Flow Adaptive	Flow Regular
2008-11-25 08:00:00	404	646	23.05	61.52	4-2	4-2	3-3	0.17	0.17	0.14
2008-11-26 05:00:00	48	33	18.52	40.74	4-2	2-4	3-3	0.07	0.17	0.15
2008-11-26 06:00:00	121	115	2.54	48.73	4-2	3-3	3-3	0.11	0.18	0.18
2008-11-26 07:00:00	348	416	8.9	54.45	4-2	3-3	3-3	0.14	0.17	0.17
2008-11-26 08:00:00	400	644	23.37	61.69	4-2	4-2	3-3	0.17	0.17	0.14
2008-11-27 05:00:00	43	34	11.69	44.16	4-2	3-3	3-3	0.09	0.16	0.16
2008-11-27 06:00:00	117	115	0.86	49.57	4-2	3-3	3-3	0.11	0.18	0.18
2008-11-27 07:00:00	304	400	13.64	56.82	4-2	3-3	3-3	0.15	0.16	0.16
2008-11-27 08:00:00	424	648	20.9	60.45	4-2	4-2	3-3	0.17	0.17	0.15
2008-11-28 05:00:00	37	35	2.78	48.61	4-2	3-3	3-3	0.11	0.18	0.18
2008-11-28 06:00:00	95	107	5.94	52.97	4-2	3-3	3-3	0.13	0.18	0.18
2008-11-28 07:00:00	284	363	12.21	56.11	4-2	3-3	3-3	0.15	0.16	0.16
2008-11-28 08:00:00	405	627	21.51	60.76	4-2	4-2	3-3	0.17	0.17	0.15
2008-12-01 05:00:00	43	44	1.15	50.57	4-2	3-3	3-3	0.12	0.18	0.18
2008-12-01 06:00:00	127	102	10.92	44.54	4-2	3-3	3-3	0.09	0.16	0.16
2008-12-01 07:00:00	277	390	16.94	58.47	4-2	4-2	3-3	0.16	0.16	0.16
2008-12-01 08:00:00	386	617	23.03	61.52	4-2	4-2	3-3	0.17	0.17	0.14
2008-12-02 05:00:00	43	34	11.69	44.16	4-2	3-3	3-3	0.09	0.16	0.16
2008-12-02 06:00:00	106	111	2.3	51.15	4-2	3-3	3-3	0.12	0.18	0.18
2008-12-02 07:00:00	310	390	11.43	55.71	4-2	3-3	3-3	0.14	0.17	0.17
2008-12-02 08:00:00	377	631	25.2	62.6	4-2	4-2	3-3	0.17	0.17	0.14
2008-12-03 05:00:00	50	32	21.95	39.02	4-2	2-4	3-3	0.07	0.17	0.14
2008-12-03 06:00:00	103	97	3.0	48.5	4-2	3-3	3-3	0.11	0.18	0.18
2008-12-03 07:00:00	278	356	12.3	56.15	4-2	3-3	3-3	0.15	0.16	0.16
2008-12-03 08:00:00	370	644	27.02	63.51	4-2	4-2	3-3	0.18	0.18	0.13
2008-12-04 05:00:00	45	44	1.12	49.44	4-2	3-3	3-3	0.11	0.18	0.18
2008-12-04 06:00:00	112	141	11.46	55.73	4-2	3-3	3-3	0.14	0.17	0.17
2008-12-04 07:00:00	313	382	9.93	54.96	4-2	3-3	3-3	0.14	0.17	0.17
2008-12-04 08:00:00	376	650	26.71	63.35	4-2	4-2	3-3	0.18	0.18	0.13
2008-12-05 05:00:00	49	38	12.64	43.68	4-2	3-3	3-3	0.08	0.16	0.16
2008-12-05 06:00:00	109	112	1.36	50.68	4-2	3-3	3-3	0.12	0.18	0.18
2008-12-05 07:00:00	288	364	11.66	55.83	4-2	3-3	3-3	0.14	0.17	0.17
2008-12-05 08:00:00	389	602	21.49	60.75	4-2	4-2	3-3	0.17	0.17	0.15
2008-12-08 05:00:00	60	32	30.43	34.78	4-2	2-4	3-3	0.04	0.18	0.12
2008-12-08 06:00:00	120	102	8.11	45.95	4-2	3-3	3-3	0.09	0.17	0.17
2008-12-08 07:00:00	298	406	15.34	57.67	4-2	4-2	3-3	0.15	0.15	0.16
2008-12-08 08:00:00	393	574	18.72	59.36	4-2	4-2	3-3	0.16	0.16	0.15
2008-12-09 05:00:00	37	37	0.0	50.0	4-2	3-3	3-3	0.12	0.18	0.18
2008-12-09 06:00:00	95	100	2.56	51.28	4-2	3-3	3-3	0.12	0.18	0.18
2008-12-09 07:00:00	275	371	14.86	57.43	4-2	4-2	3-3	0.15	0.15	0.16
2008-12-09 08:00:00	403	597	19.4	59.7	4-2	4-2	3-3	0.16	0.16	0.15

Table 1: Example of a single processed file. Data from 2008 of a sensor located in Dunmore (N77-2).

Date	Vol North	Vol South	Asymmetry	South	Reversible	Adaptive	Regular	Flow Reversible	Flow Adaptive	Flow Regular
2008-12-10 05:00:00	50	42	8.7	45.65	4-2	3-3	3-3	0.09	0.17	0.17
2008-12-10 06:00:00	113	101	5.61	47.2	4-2	3-3	3-3	0.1	0.17	0.17
2008-12-10 07:00:00	302	381	11.57	55.78	4-2	3-3	3-3	0.14	0.17	0.17
2008-12-10 08:00:00	397	615	21.54	60.77	4-2	4-2	3-3	0.17	0.17	0.15
2008-12-11 05:00:00	44	38	7.32	46.34	4-2	3-3	3-3	0.1	0.17	0.17
2008-12-11 06:00:00	112	115	1.32	50.66	4-2	3-3	3-3	0.12	0.18	0.18
2008-12-11 07:00:00	320	382	8.83	54.42	4-2	3-3	3-3	0.14	0.17	0.17
2008-12-11 08:00:00	389	658	25.69	62.85	4-2	4-2	3-3	0.17	0.17	0.14
2008-12-12 05:00:00	42	26	23.53	38.24	4-2	2-4	3-3	0.06	0.17	0.14
2008-12-12 06:00:00	111	100	5.21	47.39	4-2	3-3	3-3	0.1	0.17	0.17
2008-12-12 07:00:00	273	354	12.92	56.46	4-2	3-3	3-3	0.15	0.16	0.16
2008-12-12 08:00:00	370	587	22.68	61.34	4-2	4-2	3-3	0.17	0.17	0.14
2008-12-15 05:00:00	56	33	25.84	37.08	4-2	2-4	3-3	0.05	0.18	0.13
2008-12-15 06:00:00	113	110	1.35	49.33	4-2	3-3	3-3	0.11	0.18	0.18
2008-12-15 07:00:00	317	368	7.45	53.72	4-2	3-3	3-3	0.13	0.17	0.17
2008-12-15 08:00:00	391	642	24.3	62.15	4-2	4-2	3-3	0.17	0.17	0.14
